# A mechanistic model of in vitro plasma activation to evaluate therapeutic kallikrein-kinin system inhibitors

**DOI:** 10.1371/journal.pcbi.1012552

**Published:** 2024-11-04

**Authors:** Alireza Rezvani-Sharif, Hadi Lioe, Steven K. Dower, Matthias Pelzing, Con Panousis, Dalton J. E. Harvie, Ineke L. Muir

**Affiliations:** 1 CSL Ltd, Bio21 Institute, Melbourne, Victoria, Australia; 2 Department of Chemical Engineering, The University of Melbourne, Melbourne, Victoria, Australia; University at Buffalo - The State University of New York, UNITED STATES OF AMERICA

## Abstract

**Background:**

The kallikrein-kinin system (KKS) is a complex biochemical pathway that plays a crucial role in regulating several physiological processes, including inflammation, coagulation, and blood pressure. Dysregulation of the KKS has been associated with several pathological conditions such as hereditary angioedema (HAE), hypertension, and stroke. Developing an accurate quantitative model of the KKS may provide a better understanding of its role in health and disease and facilitate the rapid and targeted development of effective therapies for KKS-related disorders.

**Objectives:**

Here, we present a novel, detailed mechanistic model of the plasma KKS, elucidating the processes of Factor XII (FXII) activation, the kallikrein feedback loop, cleavage of high molecular weight kininogen leading to bradykinin (BK) production, and the impact of inhibitors.

**Methods:**

The model incorporates both surface and solution-phase reactions of all proteins in the KKS, describing how binding site concentration affects the rate of surface reactions. The model was calibrated and validated using a variety of published and in-house experimental datasets, which encompass a range of dextran sulphate (DXS) concentrations to initiate contact activation and various KKS inhibitors to block bradykinin production.

**Results:**

Our mathematical model showed that a trace amount of activated FXII is required for subsequent FXII activation. The model also reveals a bell-shaped curve relationship between the activation of the KKS and the number of DXS surface binding sites. Simulations of BK generation in healthy and HAE plasma demonstrated the impact of C1 esterase inhibitor (C1inh) deficiency via increased peak BK levels and accelerated formation in HAE plasma. The efficacy of KKS inhibitors, such as CSL312, ecallantide, and C1inh, was also evaluated, with CSL312 showing the most potent inhibition of BK generation.

**Conclusions:**

The present model represents a valuable framework for studying the intricate interactions within the plasma KKS and provides a better understanding of the mechanism of action of various KKS-targeted therapies.

## Author summary

The plasma kallikrein-kinin system (KKS) is a biological pathway that impacts inflammation, blood clotting, and blood pressure. This system is regulated by endogenous inhibitors; however, in diseases such as hereditary angioedema, overactivation of the plasma KKS occurs due to insufficient inhibition. The KKS operates via a series of biochemical reactions within the blood and on the surfaces of blood vessels. Each step activates an enzyme essential for the subsequent step, ultimately leading to the formation of a pro-inflammatory peptide called bradykinin. The system is initiated by the activation of Factor XII (FXII) upon contact with negatively charged surfaces. FXII activation can also occur *in vitro* by the addition of dextran sulphate (DXS). In this study, we describe an innovative mechanistic model of the plasma KKS. This model integrates both surface and volume reactions within the KKS, illustrating how variations in DXS concentration can influence reaction rates in a bell-shaped manner. The model was validated using experimental data, covering a range of DXS concentrations and clinical KKS inhibitors. We demonstrate that, in contact activation-initiated systems, targeting FXII inhibition is a more effective treatment approach than inhibiting downstream enzymes for the prevention of bradykinin generation.

## 1. Introduction

The plasma kallikrein-kinin system (KKS) is critical to cardiovascular and cerebrovascular functions and is a convergence point for several physiological processes, including the complement, coagulation, fibrinolytic, and the renin-angiotensin systems, as well as inflammatory pathways and blood pressure regulation [1,2]. The KKS comprises three serine proteases, factor XI (FXI), factor XII (FXII), and plasma prekallikrein (PK), and a nonenzymatic cofactor, high molecular weight kininogen (HK). The contact system is activated upon contact of blood with negatively-charged surfaces, initiating the slow conversion of surface-bound FXII to two-chain activated FXII (αFXIIa). In a positive feedback loop, αFXIIa activates PK to kallikrein (PKa) which reciprocally activates FXII to αFXIIa. PKa also cleaves αFXIIa to produce the single-chain activated FXII (βFXIIa) which, unlike FXII and αFXIIa, is not surface-bound. Only the αFXIIa form of activated FXII can activate FXI, initiating the intrinsic pathway of coagulation, leading to thrombin generation and clot formation. PKa hydrolyses HK, generating cleaved HK (cHK) and releasing the vasoactive, proinflammatory nona-peptide bradykinin (BK) [3].

KKS activity is regulated by α2-macroglobulin (α2M) and various members of the serpin family, including C1 esterase inhibitor (C1inh), antithrombin (AT) and α2-antiplasmin (α2AP). C1inh is the primary inhibitor of the KKS, and deficiency or dysfunction of this protein results in excessive activation of the KKS and uncontrolled production of BK. The lack of functional C1inh is the cause of hereditary angioedema (HAE) types I and II, a rare disease characterised by fluid accumulation in the deeper layers of the skin or mucosal tissues, leading to pathological swelling in areas such as the face, throat, limbs, genitals, or gastrointestinal tract. HAE attacks typically develop over a time course of several hours and can persist for two to five days if left untreated [4].

Management of HAE typically involves inhibition of the KKS. Replacement of C1inh is a common HAE treatment. Clinically available options include Berinert, Haegarda and Cinryze which are all human plasma-derived C1 esterase inhibitor and Ruconest which is a recombinant version of C1 esterase inhibitor [5,6]. Therapeutic inhibitors that target PKa such as the small molecule Berotralstat, as well as the protein-based PKa inhibitors ecallantide and lanadelumab can potentially reduce BK generation in HAE patients by impairing the KKS feedback loop [7]. CSL312 (garadacimab) is a fully human recombinant monoclonal antibody that binds to the FXII zymogen and more effectively to the active forms of FXII and inhibits PK activation by FXIIa [8]. CSL312 has been shown to be efficacious in reducing HAE attacks in a recent phase 3 clinical trial [9]. Finally, blocking the binding of BK to its receptor B2 is another HAE treatment option, which can be achieved using the synthetic peptide Icatibant [10]. A comprehensive review of various treatment options for HAE can be found in Valerieva et al. [11].

Mathematical modelling can be a convenient and informative way to evaluate the efficacy and mechanism of action of different treatments. Several studies have incorporated the contact pathway into more comprehensive models of *in vitro* blood clotting [12–16]. For instance, Kogan et al. constructed a system of time-dependent ordinary differential equations (ODEs) to predict clotting time in an activated partial thromboplastin time (aPTT) test [12]. Similarly, Kramoroff and Nigretto developed another ODE-based aPTT model and numerically optimised all kinetic parameters by fitting aPTT data for normal and single-factor-deficient plasma [15]. Chatterjee et al. used a detailed ODE model of coagulation and demonstrated that even in the presence of excess corn trypsin inhibitor (an inhibitor of FXIIa), trace amounts of uninhibited FXIIa are sufficient to trigger a coagulation response [16]. However, in these models, no surface binding reactions were included, HK was omitted, and often no distinction was made between αFXIIa and βFXIIa.

To the best of our knowledge, no mathematical model has been developed specifically for the KKS. The main challenge in developing such a model is the crucial dependency of the system on surface-mediated reactions. The type and concentration of negatively-charged surfaces have a significant impact on the activity of the contact activation system [17]. In this study, we introduce a mechanistic model of BK generation via the KKS. The model accounts for surface-binding reactions and describes the effect of surface-site concentration on the apparent rate of these reactions. The model has been validated using published experimental datasets. It enables the estimation of C1inh deficiency effects on BK production levels and provides a comprehensive analysis of the mechanism of action of several KKS inhibitors on BK formation under static (non-flow) conditions.

## 2. Material and methods

### 2.1. Reaction scheme

**Fig 1:**
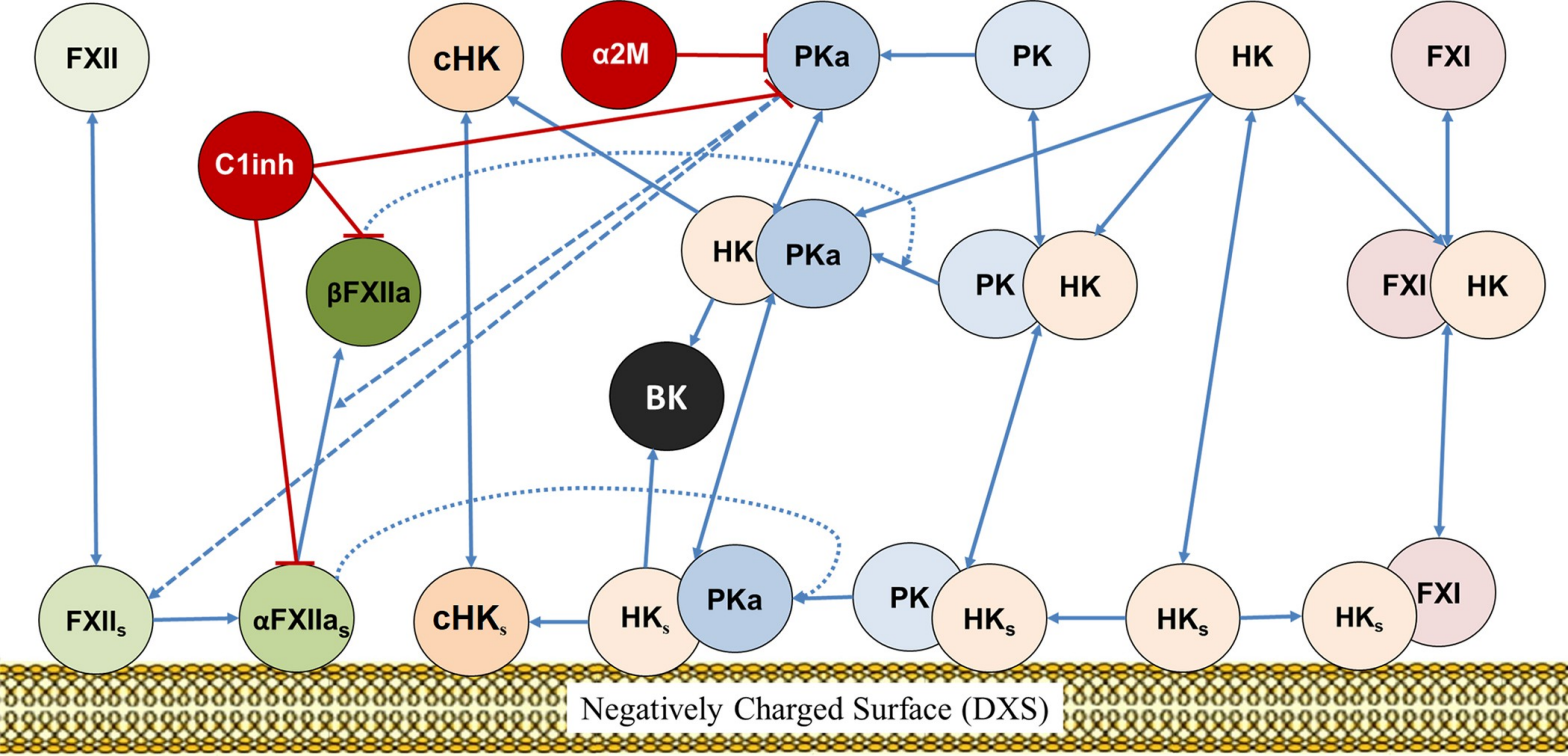
BK generation reaction network including surface-mediated reactions. FXII: factor FXII, αFXIIa: two-chain activated factor FXII, βFXIIa: single-chain activated FXII, FXI: factor FXI, PK: prekallikrein, PKa: kallikrein, HK: high molecular weight kininogen, cHK: cleaved high molecular weight kininogen, BK: bradykinin, α2M: α2-macroglobulin, C1inh: C1 esterase inhibitor. Surface-bound species are indicated by subscript ‘s’.

Fig 1 shows a simplified KKS reaction scheme and provides information on the interactions between proteins, including those that bind to negatively-charged surfaces. S1 Table lists the species, their molecular weights, and initial concentrations in the KKS model. The biochemical reactions and their corresponding kinetic parameters are outlined in Table 1. S1 Fig represents the comprehensive scheme of the plasma KKS network model corresponding to Table 1. The KKS reactions can be classified into six categories, which are described below.

**Table 1:** List of biochemical reactions in the computational model of the kallikrein-kinin system. Surface-bound species are indicated by subscript ‘s’, and solution-phase species are indicated by subscript ‘v’.

	Reaction	k_a_ (M^-1^s^-1^)	k_d_ (s^-1^)	K_D_ (nM)	k_cat_ (s^-1^)	K_m_ or K_D_ (nM)	k_cat_ (s^-1^)	Ref
		Model value				Literature range		
1	FXII_v_ + S_s_ <-> (FXII-S)_s_	10^8^ ^a^	100	1000	-	170–1000	-	[22, 23]
2	αFXIIa_v_ + S_s_ <-> (αFXIIa-S)_s_	10^8^	17	170				
3	(FXII-S)_s_ -> (αFXIIa-S)_s_ ^b^	k = 1.6х10^−4^ s^-1^				k = 1.6–5х10^-4^s^-1^		[16, 22]
4	(FXII-S)_s_ + (αFXIIa-S)_s_ <-> (FXII-S-αFXIIa-S)_s_ -> (αFXIIa-S)_s_ + (αFXIIa-S)_s_	1.5х[DXS]^-1^ х10^7^ ^c^	1.1	73.3х [DXS] ^c^	0.033	7500	0.033	[24]
5	(αFXIIa-S)_s_ + (αFXIIa-S)_s_ <-> (αFXIIa-S-αFXIIa-S)_s_ -> (αFXIIa-S)_s_ + βFXIIa + S_s_	1.5х[DXS]^-1^ х10^7^ ^c^	1.1	73.3х [DXS] ^c^	0.033	No data^d^		
6	FXI_v_ + HK_v_ <-> (FXI-HK)_v_	10^8^	1.9	19	-	2.4–69	-	[52–54]
7	FXI_v_ + cHK_v_ <-> (FXI-cHK)_v_							
8	FXI_v_ + (HK-S)_s_ <-> (FXI-HK-S)_s_							
9	FXI_v_ + (cHK-S)_s_ <-> (FXI-cHK-S)_s_							
10	PK_v_ + HK_v_ <-> (PK-HK)_v_	10^8^	1	10	-	10–29	-	[52–54]
11	PK_v_ + cHK_v_ <-> (PK-cHK)_v_							
12	PK_v_ + (HK-S)_s_ <-> (PK- HK-S)_s_							
13	PK_v_ + (cHK-S)_s_ <-> (PK-cHK-S)_s_							
14	PKa_v_ + cHK_v_ <-> (PKa-cHK)_v_	10^8^	9	90	-	90	-	[55]
15	PKa_v_ + (cHK-S)_s_ <-> (PKa-cHK-S)_s_							
16	(PKa-C1inh)_v_ + cHK_v_ <-> (PKa-C1inh-cHK)_v_							
17	(PKa-C1inh)_v_ + (cHK-S)_s_ <-> (PKa-C1inh-cHK-S)_s_							
18	(PKa-AT)_v_ + cHK_v_ <-> (PKa-AT-cHK)_v_							
19	(PKa-AT)_v_ + (cHK-S)_s_ <-> (PKa-AT-cHK-S)_s_							
20	HK_v_ + 1.5S_s_ <-> (HK-S)_s_	10^8^	100	1000	-	No data ^e^		
21	cHK_v_ + 1.5S_s_ <-> (cHK-S)_s_							
22	(FXI-HK)_v_ + 1.5S_s_ <-> (FXI-HK-S)_s_							
23	(FXI-cHK)_v_ + 1.5S_s_ <-> (FXI-cHK-S)_s_							
24	(PK-HK)_v_ + 1.5S_s_ <-> (PK-HK-S)_s_							
25	(PK-cHK)_v_ + 1.5S_s_ <-> (PK-cHK-S)_s_							
26	(PKa-HK)_v_ + 1.5S_s_ <-> (PKa-HK-S)_s_							
27	(PKa-cHK)_v_ + 1.5S_s_ <-> (PKa-cHK-S)_s_							
28	PK_v_ + (αFXIIa-S)_s_ <-> (PK-αFXIIa-S)_s_ -> PKa_v_ + (αFXIIa-S)_s_	10^8^	28.4	291	0.7	91–170	0.7–3.6	[24, 56]
29	(PK-HK-S)_s_ + (αFXIIa-S)_s_ <-> (PK-HK-S-αFXIIa-S)_s_ -> (PKa-HK-S)_s_ + (αFXIIa-S)_s_	[DXS]^-1^х10^7 c^	16.3	2330х [DXS] ^c^	3.6	91	3.6	[24]
30	(PK-cHK-S)_s_ + (αFXIIa-S)_s_ <-> (PK-cHK-S-αFXIIa-S)_s_ -> (PKa-cHK-S)_s_ + (αFXIIa-S)_s_	[DXS]^-1^х10^7 c^	16.3	2330х [DXS] ^c^	3.6			
31	PK_v_ + βFXIIa_v_ <-> (PK-βFXIIa)_v_ -> PKa_v_ + βFXIIa_v_	10^6^	36.6	37000	40	37000	40	[24]
32	(PK-HK)_v_ + βFXIIa_v_ <-> (PK-HK-βFXIIa)_v_ -> (PKa-HK)_v_ + βFXIIa_v_							
33	(PK-cHK)_v_ + βFXIIa_v_ <-> (PK-cHK-βFXIIa)_v_ -> (PKa-cHK)_v_ + βFXIIa_v_							
34	FXII_v_ + PKa_v_ <-> (FXII-PKa)_v_ -> αFXIIa_v_ + PKa_v_	10^6^	11	11000	0.01	11000	0.01	[24]
35	FXII_v_ + (PKa-HK)_v_ <-> (FXII-PKa-HK)_v_ -> αFXII_v_ + (PKa-HK)_v_							
36	FXII_v_ + (PKa-HK)_v_ ⬄ (FXII-PKa-HK)_v_ -> αFXII_v_ + (PKa-HK)_v_							
37	(FXII-S)_s_ + (PKa-HK-S)_s_ <-> (FXII-PKa-HK-S)_s_ -> (αFXIIa-S)_s_ + (PKa-HK-S)_s_	5х[DXS]^-1^ х10^8 c^	1.1	13.2 х [DXS] ^c^	5.7	11.7–510	5.7	[24]
38	(FXII-S)_s_ + (PKa-cHK-S)_s_ <-> (FXII-PKa-cHK-S)_s_ -> (αFXIIa-S)_s_ + (PKa-cHK-S)_s_							[24]
39	(αFXIIa-S)_s_ + (PKa-HK-S)_s_ <-> (αFXIIa-S-PKa-HK-S)_s_ -> βFXIIa_v_ + S_s_ + (PKa-HK-S)_s_	5х[DXS]^-1^ х10^8 c^	45.3	462	5.7	No data^d^		[24]
40	(αFXIIa-S)_s_ + (PKa-cHK-S)_s_ <-> (αFXIIa-S-PKa-cHK-S)_s_ -> βFXIIa_v_ + S_s_ + (PKa-cHK-S)_s_							
41	(PK-HK-S)_s_ + (PKa-HK-S)_s_ -> (PKa-HK-S)_s_ + (PKa-HK-S)_s_	k = 2.7 х [DXS]^-1^х10^4^ M^-1^ s^-1 c^				k = 2.7х10^4^ M^-1^ s^-1^		[57]
42	(PK-cHK-S)_s_ + (PKa-HK-S)_s_ -> (PKa-cHK-S)_s_ + (PKa-HK-S)_s_							
43	(PK-HK-S)_s_ + (PKa-cHK-S)_s_ -> (PKa-HK-S)_s_ + (PKa-cHK-S)_s_							
44	(PK-cHK-S)_s_ + (PKa-cHK-S)_s_ -> (PKa-cHK-S)_s_ + (PKa-cHK-S)_s_							
45	PKa_v_ + (HK-S)_s_ <-> (PKa-HK-S)_s_ -> (PKa-cHK-S)_s_ + BK_v_	10^8^	12.6	127.2	1.2	139–1260	1.2	[41, 58]
46	(PKa-C1inh)_v_ + (HK-S)_s_ <-> (PKa-C1inh-HK-S)_s_				-		-	
47	(PKa-AT)_v_ + (HK-S)_s_ <-> (PKa-AT-HK-S)_s_							
48	PKa_v_ + HK_v_ <-> (PKa-HK)_v_ -> (PKa-cHK)_v_ + BK_v_	10^8^	44	440	0.63	440–1380	0.007–0.63	[41, 58]
49	(PKa-C1inh)_v_ + HK_v_ <-> (PKa-C1inh-HK)_v_				-		-	
50	(PKa-AT)_v_ + HK_v_ <-> (PKa-AT-HK)_v_							
51	BK_v_ -> BK_deg_v_ ^f^	K = 0.046 s^-1^				K = 0.046 s^-1^		[40]
52	αFXIIa_v_ + C1inh_v_ -> αFXIIa-C1inh_v_ ^g^	k = 3.6х10^3^ M^-1^ s^-1^				k = 3.6х10^3^ M^-1^ s^-1^		[36]
53	αFXIIa_v_ + α2AP_v_ -> αFXIIa- α2AP_v_	k = 180 M^-1^ s^-1^				k = 180 M^-1^ s^-1^		[36]
54	(αFXIIa-S)_s_ + α2AP_v_ -> (αFXIIa-S-α2AP)_s_							
55	αFXIIa_v_ + α2M_v_ -> αFXIIa-α2M_v_	k = 83.3 M^-1^ s^-1^				k = 83.3 M^-1^ s^-1^		[36]
56	(αFXIIa-S)_s_ + α2M_v_ -> (αFXIIa-S-α2M)_s_							
57	αFXIIa_v_ + AT_v_ -> αFXIIa-AT_v_	k = 22 M^-1^ s^-1^				k = 22 M^-1^ s^-1^		[36]
58	(αFXIIa-S)_s_ + AT_v_ -> (αFXIIa-S-AT)_s_							
59	βFXIIa_v_ + C1inh_v_ -> βFXIIa-C1inh_v_	k = 3.1х10^3^ M^-1^ s^-1^				k = 3.1х10^3^ M^-1^ s^-1^		[49]
60	βFXIIa_v_ + α2AP_v_ -> βFXIIa- α2AP_v_	k = 150 M^-1^ s^-1^				k = 150 M^-1^ s^-1^		[49]
61	βFXIIa_v_ + AT_v_ -> βFXIIa- AT_v_	k = 53 M^-1^ s^-1^				k = 53 M^-1^ s^-1^		[49]
62	PKa_v_ + C1inh_v_ -> PKa-C1inh_v_	k = 17 х10^3^ M^-1^ s^-1^				k = 1.7–4.5х10^4^ M^-1^s^-1^		[16, 51]
63	(PKa-HK)_v_ + C1inh_v_ -> (PKa-C1inh-HK)_v_							
64	(PKa-cHK)_v_ + C1inh_v_ -> (PKa-C1inh-cHK)_v_							
65	(PKa-HK-S)_s_ + C1inh_v_ -> (PKa-C1inh-HK-S)_s_							
66	(PKa-cHK-S)_s_ + C1inh_v_ -> (PKa-C1inh-cHK-S)_s_							
67	PKa_v_ + α2M_v_ -> PKa-α2M_v_ ^h^	k = 5.8х10^3^ M^-1^ s^-1^				k = 5.8–34 х10^3^ M^-1^s^-1^		[37, 50]
68	PKa_v_ + AT_v_ -> (PKa-AT)_v_	k = 240 M^-1^ s^-1^				k = 240 M^-1^ s^-1^		[59]
69	(PKa-HK)_v_ + AT_v_ -> (PKa-HK-AT)_v_							
70	(PKa-HK-S)_s_ + AT_v_ -> (PKa-HK-S-AT)_s_							
71	(PKa-cHK)_v_ + AT_v_ -> (PKa-cHK-AT)_v_							
72	(PKa-cHK-S)_s_ + AT_v_ -> (PKa-cHK-S- AT)_s_							

a On-rates were assumed to be diffusion limited (k_a_ = 1х10^8^ M^-1^s^-1^) [16].

^b^ FXII auto-activation was considered only in the Section 3.1.

^c^ In the forward rate of surface-mediated reactions, [DXS] should be expressed in nM.

^d^ No kinetic data is available for the conversion rate of αFXIIa to βFXIIa; thus, it was assumed to be equal to the rate of FXIIa conversion to αFXIIa.

e Although the binding of HK to endothelial cells and platelets has been studied extensively (KD = 7–98 nM), there is no data available on HK/cHK binding affinity to DXS.

f The BK degradation reaction was considered only in Section 3.3.

g Surface-bound αFXIIa is protected from inhibition by C1inh [60].

h HK protects PKa from inhibition by α2M.

#### 2.1.1. Surface binding of FXII and HK

Contact activation can be triggered via the interaction of FXII with negatively-charged physiological activators such as heparin, neutrophil extracellular traps (NETs), plasma microparticles, collagen, misfolded protein aggregates, and nucleic acids [18]. It has been suggested that platelets, along with platelet-secreted polyphosphates (poly-P) and platelet-derived microparticles, can serve as negatively charge surfaces upon which FXII autoactivates. However, recent studies have questioned the effectiveness of platelet-derived poly-P in FXII activation due to their insufficient chain length [19]. FXII can also bind to negatively-charged artificial surfaces such as kaolin and dextran sulphate (DXS) in a concentration-dependent manner [20], undergoing conformational changes to become autoactivated. The N-terminal of the heavy-chain region of FXII contains at least two binding sites for negatively-charged surfaces [21].

DXS is a known potent activator of the contact pathway and has been extensively utilised to initiate FXII activation [22–25]. The number of FXII binding sites on DXS molecules depends on the (average) molecular weight of the polymeric DXS chains. There is consensus in the literature that contact activation cannot be initiated by DXS with a mean molecular weight below 3–5 kDa [25,26]. This paper focuses on the activation of the contact system via DXS with a mean molecular weight of 500 kDa since this polymeric chain length has been extensively studied in the context of FXII activation, due to its ability to effectively initiate FXII autoactivation, enhance FXII susceptibility for cleavage by PKa, and trigger KKS activation in plasma [25]. Different values for the binding affinity and number of binding sites for FXII on 500 kDa DXS have been reported [22,23]. Samuel et al. suggested that 165–192 FXII molecules can bind to one such DXS chain with an affinity of 170 nM [23]. Loiseau et al. provided an estimate of 220 FXII molecules per 500 kDa DXS chain but reported a much lower affinity with a *K*_*d*_ of 1 μM [22].

Like FXII, HK and cHK also contain binding regions for both artificial and biological negatively-charged surfaces [27]. A wide range of affinities, from low nanomolar to low micromolar values, along with a varying number of binding sites for HK on endothelial cells, have been reported in the literature. However, no kinetic data on the binding of HK/cHK to DXS was found. Considering the molecular weights (relative sizes) of HK and FXII, we assumed that the number of binding sites per DXS chain for FXII is two-thirds of that for HK. We also posited that when FXII or HK form complexes with other species, these complexes occupy the same binding site size on DXS as for non-complex FXII or HK. Given that proteins forming complexes with FXII and HK lack surface binding domains, they are presumed not to obscure a significant amount of DXS when complexed with surface-bound FXII or HK.

#### 2.1.2. Complexation of PK, PKa and FXI with HK(a)

PK, PKa, and FXI do not bind directly to surfaces, but instead form complexes with HK which is able to bind negatively-charged surfaces. The binding sites for PK and FXI on HK overlap, such that HK cannot bind both PK and FXI simultaneously [3]. Nevertheless, since HK is present in plasma in considerable molar excess, 75–80% of PK and 95% of FXI are bound to HK *in vivo* [28]. To account for the competitive binding of FXI to HK, we added FXI to the model, but ignored the activation of FXI by αFXIIa, as there is no evidence for FXII-dependent activation of FXI in the presence of DXS [29].

There is inconsistency in the literature regarding the affinity of PKa for HK. Some studies suggest that the affinity of PKa for HK is about the same as that of PK for HK [30], while others report a much lower affinity [31]. In this study, we explored both possibilities through model optimisation, and the results suggested that the affinity of PKa for HK is indeed lower than the affinity of PK for HK.

#### 2.1.3. FXII activation

**Fig 2:**
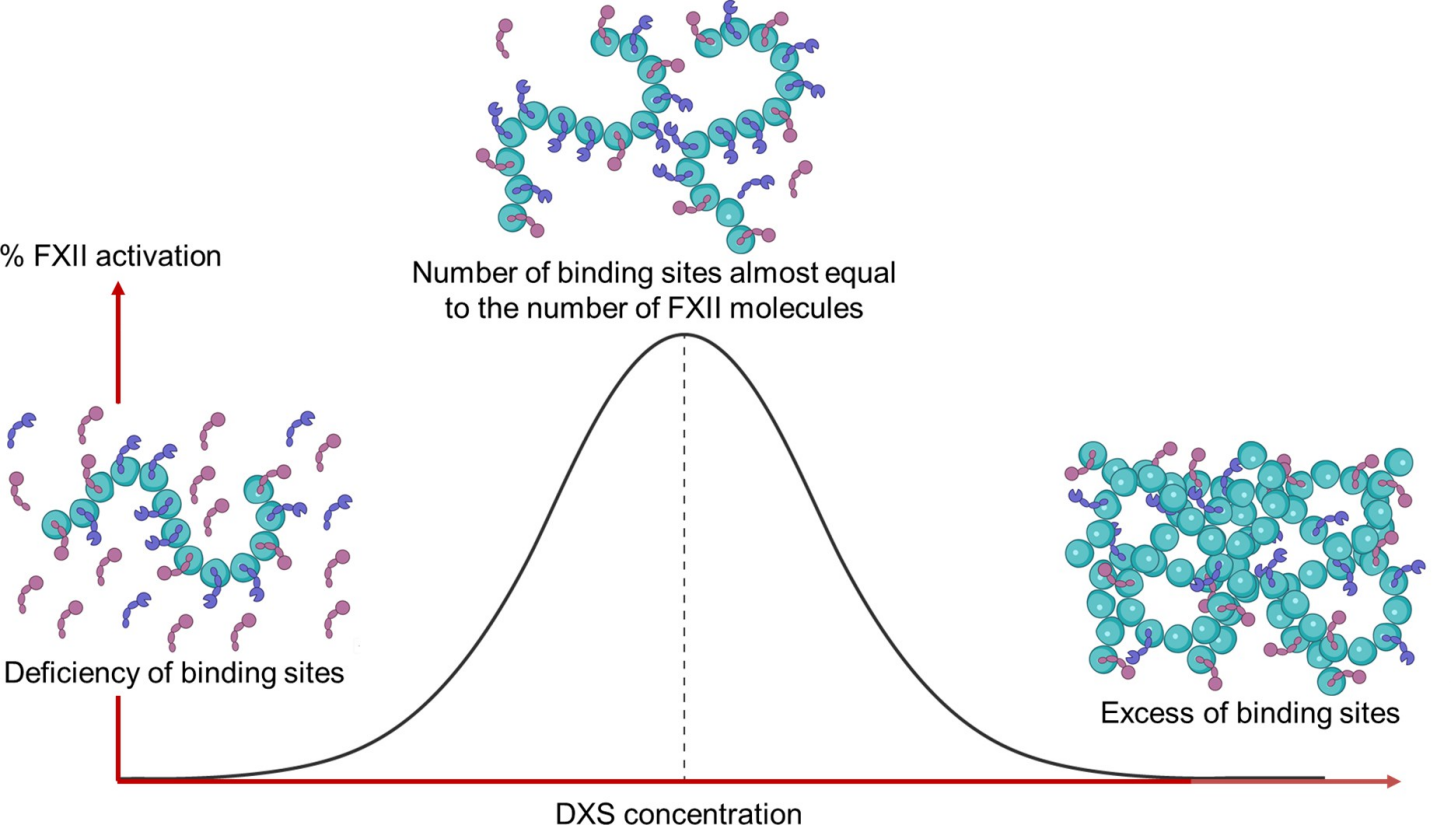
Effect of DXS concentration on the rate of FXII activation.

The rate equations describing the conversion of FXII to its active, enzymatic form need to include the (DXS) surface-binding reactions. It has been established that FXII needs to bind to negatively charged surfaces to undergo autoactivation, during which the surface-bound FXII zymogen transforms into αFXIIa. Subsequently, the newly formed surface-bound αFXIIa can activate an adjacent surface-bound FXII zymogen, suggesting that FXII self-activation also proceeds on surfaces [32]. Further evidence of the necessity of surface-binding sites comes from the observation that the apparent rate of FXII activation depends on the concentration of DXS via a bell-shaped curve and passes through a maximum as the total activator concentration is increased [22,23,25]. For instance, Loiseau et al. [22] incubated 350 nM FXII with various concentrations of 500 kDa DXS and showed that maximum FXII activation rate occurs in the presence of about 6 nM DXS. As shown in Fig 2, at low surface-site (DXS) concentrations (i.e., when there is a molar excess of FXII), the apparent FXII activation rate is limited by the availability of binding sites. As the number of binding sites rises (with increasing DXS concentration), the proportion of surface-bound FXII and αFXIIa increases, and the rate of FXII self-activation will be maximal. However, further increasing the DXS concentration results in an excess of available binding sites, which leads to a decrease in the surface concentration of FXII and FXIIa, ultimately reducing the rate of FXII activation.

The reactions describing FXII activation on DXS are listed below. Reactions 1 and 2 describe the surface binding equilibria for FXII and αFXIIa, respectively. Surface-bound species are indicated by subscript ‘s’ and those in the solution by subscript ‘v’. S_s_ represents the concentration of binding sites for FXII or αFXIIa on a DXS molecule, noting that the sites for these two different species are assumed identical. The initiation of contact activation is often attributed to either the autoactivation of surface-bound FXII (Reaction 3) or the presence of a trace amount of αFXIIa in plasma that results in FXII self-activation (Reaction 4), or a combination of both processes. Reaction 5 represents the formation of βFXIIa, which does not bind to surfaces and lacks coagulant activity and the capability to further cleave factor XII.

$$FXII_{v}+S_{s}\overset{k_{a\_1}}{\underset{k_{d\_1}}{\Leftrightarrow }}{\left(FXII:S\right)}_{s}$$ (1)

$$\alpha FXIIa_{v}+S_{s}\overset{k_{a\_2}}{\underset{k_{d\_2}}{\Leftrightarrow }}{\left(\alpha FXIIa:S\right)}_{s}$$ (2)

$$FXII_{s}\overset{k_{a\_3}}{\Rightarrow }\alpha FXIIa_{s}$$ (3)

$${\left(FXII:S\right)}_{s}+{\left(\alpha FXIIa:S\right)}_{s}\overset{k_{a\_4}}{\underset{k_{d\_4}}{\Leftrightarrow }}{\left(FXII:S:\alpha FXIIa:S\right)}_{s}\overset{k_{cat\_4}}{\Rightarrow }2{\left(\alpha FXIIa:S\right)}_{s}$$ (4)

$${\left(\alpha FXII:S\right)}_{s}+{\left(\alpha FXIIa:S\right)}_{s}\overset{k_{a\_5}}{\underset{k_{d\_5}}{\Leftrightarrow }}{\left(\alpha FXII:S:\alpha FXIIa:S\right)}_{s}\overset{k_{cat\_5}}{\Rightarrow }{\left(\alpha FXIIa:S\right)}_{s}+\beta FXIIa_{v}+S_{s}$$ (5)

As detailed in the S1 Text, when two surface-bound species interact, as observed in the reversible part of reaction 4 and in reaction 5, the effective forward rate constant changes with the concentration of DXS. For example, the effective forward rate for reaction 4 can be expressed as:

$$R_{4v,i}=\frac{\tilde{k_{a\_4}}}{\left[DXS\right]}[{\left(FXII:S\right)}_{s}][{\left(\alpha FXIIa:S\right)}_{s}]-k_{d\_4}[({FXII:S-\alpha FXIIa:S)}_{s}]$$ (6)

where *R*_4*v*,*i*_ denotes the volumetric reaction rate with units of M. s^-1^, $\frac{\tilde{k_{a\_4}}}{\left[DXS\right]}$ represents the forward rate with units of M^-1^. s^-1^, and $\tilde{k_{a\_4}}$ is a constant that is dependent on the surface area of each polymer chain, which in turn is related to the molecule weight of DXS.

More recently, studies have demonstrated that the rate of FXII activation by PKa-HK follows a similar bell-shaped curve in response to changes in DXS concentration, due to competitive binding between FXII and PKa-HK for DXS [33]. Therefore, a modelling approach similar to the one explained above was employed to describe FXII activation by surface-bound PKa-HK [25,33,34]. By reviewing the DXS concentrations used in published studies that report kinetic parameters for these surface reactions, we were able to estimate initial values for the effective forward rate at various DXS levels. Subsequently, we conducted model calibration using different datasets to determine the optimised values.

#### 2.1.4. PK activation

It has been shown that the PK-HK complex, when bound to a surface, is activated by surface-bound PKa-HK. However, this mechanism is unlikely to be a physiologically significant mechanism of PKa generation except possibly during the initiation phase of contact activation, when the concentration of FXIIa is low [35].

It is well established that surface-bound PK-HK complex is activated by surface-bound αFXIIa, and fluid-phase PK is activated by βFXIIa [24]. We have considered a similar bell-shaped curve dependency on the availability of DXS surface sites for both the activation rate of surface-bound PK-HK by αFXIIa and the self-activation of PK, comparable to the activation of FXII on DXS.

#### 2.1.5. Inhibition of FXIIa and PKa

In normal plasma, C1inh is the only major inhibitor of αXIIa and βXIIa [36]. However, in HAE patients, inhibitors that are typically of minor significance, such as α2M, α2AP, and AT, assume greater importance [36]. The primary inhibitors of PKa in normal plasma are C1inh and α2M [37], but in HAE patients, the roles of α2M and AT as PKa inhibitors are accentuated [38,39]. All inhibitors of FXIIa and PKa exhibit 1:1 stoichiometric binding with their respective enzymes [31,36].

#### 2.1.6. BK formation and degradation

BK is a vasoactive nonapeptide which is cleaved from HK through the enzymatic action of PKa. Despite its critical roles, BK is a transient species in plasma with an extremely short half-life of approximately 15 seconds [40], due to its rapid degradation in the circulatory system. The catalytic efficiency of BK release from HK by PKa is slightly higher in the presence of DXS. This facilitation is thought to be due to the conformational changes induced in the substrate HK by DXS [41].

### 2.2. Mathematical modelling

#### 2.2.1. Model formulation

Biochemical reactions listed in Table 1 were converted into a system of ODEs using the laws of mass action kinetics as shown in S2 Table. Numerical simulations were conducted using arb [42], an open-source package that solves ODEs via a back-stepped Newton-Raphson scheme. Initial values for the concentrations of species and rate constants were extracted from the literature (Tables 1 and S1). When different values were reported for a rate constant, the mean of the range was considered as the initial value for sensitivity analysis and model calibration.

#### 2.2.2. Sensitivity analysis

Sensitivity analysis was conducted to assess the impact of uncertainties associated with both the initial concentrations of species and the rate constants on model outputs. As depicted in S2 Fig, BK generation displays three phases in our closed model system: initiation, propagation, and termination. For sensitivity analysis, we focused on two parameters: the time required to generate 20 nM of BK, and the peak BK level. We individually perturbed the initial concentrations of species and the rate constants, varying them from 10% to 1000% of the baseline model values. This range was selected to encompass the variability observed in these parameters as reported in the literature. The baseline model value was set at 100%, and the changes in the model output by perturbing each parameter were compared with the baseline model. The outcomes of the sensitivity analysis are presented in S3 and S4 Figs.

#### 2.2.3. Model calibration and validation

Model calibration and optimisation were undertaken to identify the sets of parameters that provided the best fit to the experimental data [8,29,43]. During the optimisation process, uncertainty ranges from 50% to 200% were considered for all kinetic parameters in the model. When different literature values were found for a kinetic parameter, we varied the parameter over the range which spanned all published values for parameter optimisation. However, the initial concentrations of species were kept constant. Optimised values were calculated using a descent method that minimised the root mean square (RMS) difference between simulated and experimental results.

Initially, we focused on the activation of pure FXII through a range of 500 kDa DXS concentrations to elucidate the specific mechanism triggering contact activation. In this step, we assessed the accuracy of our equations that describe the bell-shaped response of FXII activation at varying DXS concentrations. Additionally, we calibrated the rate of FXII self-activation, the binding kinetics of FXII to DXS, and the number of FXII binding sites on the DXS chain.

Once these parameters were calibrated to fit experimental data of purified FXII solutions, we considered DXS activation of FXII in plasma, using a DXS concentration range spanning four orders of magnitude. In this step, all the kinetic parameters, excluding those calibrated in the previous step, were fine-tuned to accurately represent the activation of FXII and PK, as well as the consumption of HK at various DXS concentrations. Subsequently, the model was validated by examining BK levels produced in the presence of three different KKS inhibitors: CSL312, lanadelumab, and C1inh, each targeting a specific pathway in BK formation. CSL312 binds to the FXII zymogen and more effectively to the active forms of FXII and inhibits PK activation by FXIIa. Lanadelumab specifically inhibit PKa and prevent the cleavage of HK. C1inh is a broader inhibitor that binds to both FXIIa and PKa; however, its potency is significantly lower compared to the others.

#### 2.2.4. Model predictions

Using the validated model, we conducted predictive analyses. These included simulating BK generation in both healthy and HAE plasma, investigating the impact of various DXS concentrations on BK levels, and comparing CSL312 with a hypothetical antibody capable of binding to both PK and PKa.

### 2.3. LC-MS/MS experiments

#### 2.3.1. Materials

Dextran Sulfate (average molecular weight > 500,000 Da) was purchased from Sigma Aldrich (product number: D6001). [Sar^0^, D-Phe^8^, Des-Arg^9^] Bradykinin (Sar-BK) was purchased from Phoenix Pharmaceuticals. [^2^H_5_-D-Phe5]Bradykinin (BK*) and [^2^H_5_-D-Phe5]Bradykinin (1–5) (BK_1-5_*) were synthesized and purified in house.

#### 2.3.2. In vitro experiments

An *in vitro* plasma experiment coupled with sensitive liquid chromatography tandem mass spectrometry (LC-MS/MS)-based assay with high specificity for quantifying BK generation in DXS-activated plasma had been developed in house. This assay can detect and quantify BK and all its major breakdown products in parallel. All in vitro plasma experiments were performed using Eppendorf Thermomixer C sample incubator and shaker, and all LC-MS/MS experiments were performed using Sciex QTRAP 6500 triple quadrupole mass spectrometer coupled with an Agilent 1290 HPLC system.

To measure the concentration of BK in plasma samples for kinetics experiments (Section 3.2.2), 100 μL of a protease inhibitor mixture was added to 1.0 mL of a frozen plasma aliquot. This mixture consisted of 50 μM (final concentration) each of Captopril, Diprotin A, Plummer’s Inhibitor, and Bestatin. The plasma sample was allowed to thaw slowly to room temperature, then centrifuged at 3,000 rpm for 5 minutes. Subsequently, 900 μL of the supernatant was collected and pre-incubated at 37°C for 5 minutes. To the plasma sample, 90 μL of Internal standard (Sar-BK) and 90 μL of DXS (final concentration = 100 μg/mL) were added, and the mixture was immediately vortexed, gently centrifuged, and incubated at 37°C. A small aliquot (40 μL) of the plasma mixture was aspirated at different time points (from 0.5 to 60 minutes) and mixed with a 3x excess of ice-cold methanol to precipitate proteins and extract BK and related species. The mixture was vortexed, left on ice for one hour to ensure complete precipitation and centrifuged at 13,000 rpm for 10 minutes. A small amount of the supernatant (30 μL) was diluted and mixed with 120 μL of 0.1% acetic acid in water and the mixture analysed by LC-MS/MS for quantitation.

For measurement of BK concentration in DXS-activated plasma in the presence of different KKS inhibitor (Section 3.4), please refer to our previous manuscript for more details [8].

## 3. Results

### 3.1. Modelling the effect of DXS concentration on FXIIa generation via either auto- or self-activation of FXII

Experimental data from Røjkjær and Schousboe [44] was used to calibrate the rates of FXII activation from reactions 1 to 5, and to find the number of FXII binding sites per 500 kDa DXS chain. Røjkjær and Schousboe [44] incubated 250 nM FXII over a range of DXS concentrations for 30 minutes, in the absence of zinc ions and measured the level of FXIIa generation via a chromogenic substrate. For more detailed information about the experimental procedures, the reader is referred to the paper by Røjkjær and Schousboe [44].

**Fig 3:**
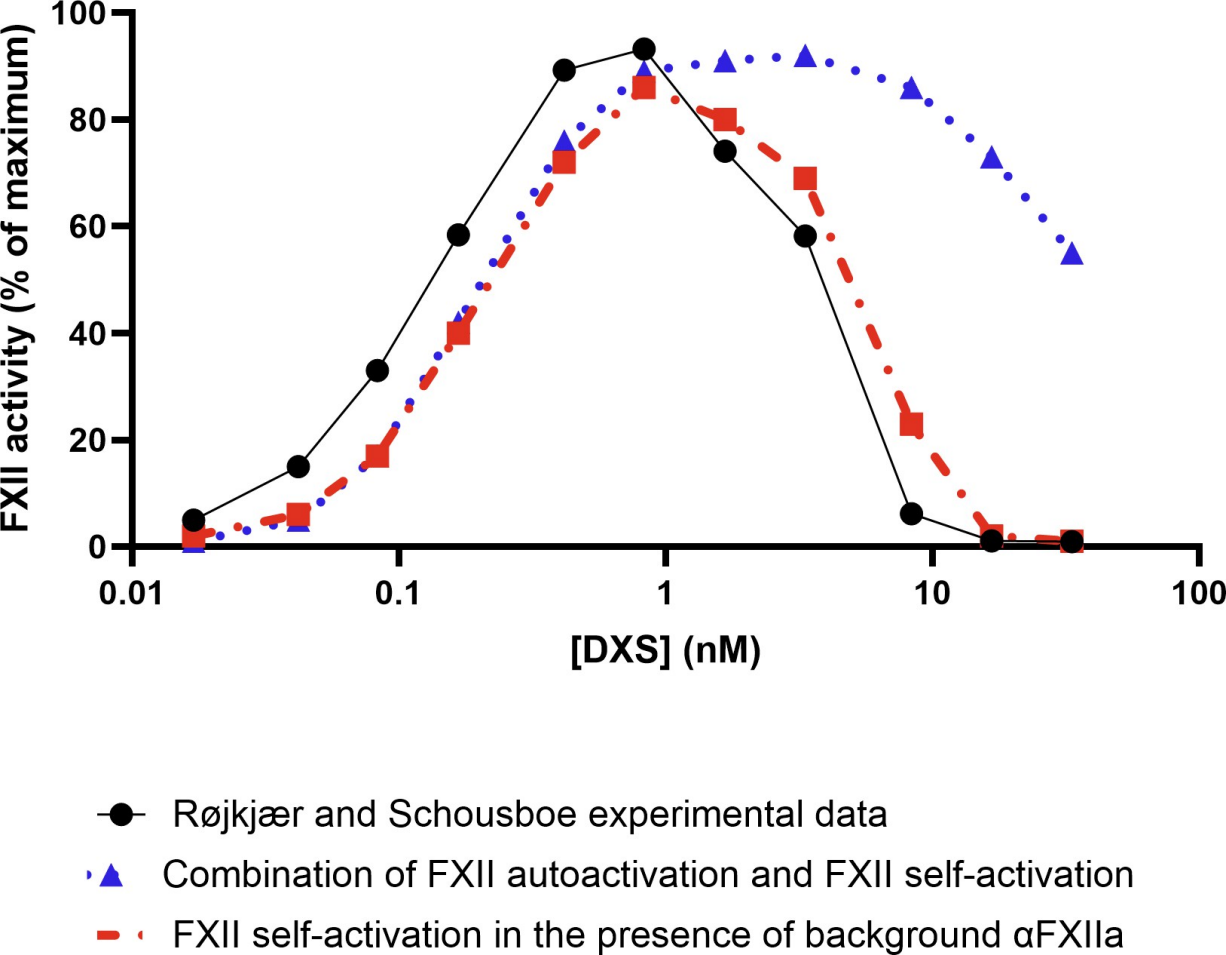
FXII activation versus DXS concentration via two alternative FXII activation mechanisms. Comparison of the Røjkjær and Schousboe [44] experimental data with simulations using optimised kinetic parameters for FXII activation, via either a combination of FXII autoactivation (reaction 3) and FXII self-activation (reaction 4), or just FXII self-activation in the presence of background αFXIIa.

As a part of the model calibration procedure, we evaluated two mechanisms for FXII activation. For the first, contact activation occurs spontaneously upon binding of FXII to DXS and then proceeds via a combination of both autoactivation and self-activation mechanisms. For the second, FXII does not activate spontaneously; instead, a small amount of systemic αFXIIa, which is already circulating in the blood, binds to an activating surface and then generates further FXIIa exclusively through self-activation. To investigate the second possibility, we eliminated the autoactivation reaction (Reaction 3) from the model. We then adjusted the initial αFXIIa concentration to fit the experimental observations reported by Røjkjær and Schousboe [44]. This calibration process assumed that initial αFXIIa levels ranges from 0.02% to 0.7% of total FXII [45], while the rate of autoactivation in the first scenario was assumed to vary between 1.6х10^−4^ s^-1^ [22] to 5х10^−4^ s^-1^ [16]. As shown in Fig 3, both hypothesized mechanisms of FXII activation were able to successfully describe FXII activation at low concentrations of DXS, however, at high DXS concentrations, the model incorporating both autoactivation and self-activation failed to accurately predict the observed level of FXII activation. This discrepancy can be attributed to the continuous generation of FXIIa by the autoactivation mechanism, which does not align with the experimental data.

The optimisation procedure determined that each DXS chain contains 220 FXII binding sites. This finding is consistent with the range of FXII binding sites per DXS chain reported in the literature [22,25]. The optimised kinetic values for these reactions are listed in Table 1, which have been applied to all subsequent simulations. Notably, during the optimisation process, we determined $\tilde{k_{a\_4}}$ for the FXII self-activation reaction. As discussed in Section 2.1.3, the actual on-rate for the self-activation reaction varies as a function of DXS concentration.

#### 3.1.1. Activation of the KKS within pooled plasma using different concentrations of DXS

Plasma activation was simulated at both low and high concentrations of 500 kDa DXS. During these simulations, we determined rate constants for the activation of the PK-HK complex by surface-bound αFXIIa, as well as for the activation of FXII by the PKa-HK complex, using a model calibration approach analogous to that employed for the FXII self-activation reaction. Consistent with the findings from the previous section, we assumed that the initial level of αFXIIa is 0.1% of total FXII across all simulations, and that FXII autoactivation does not occur. We also assumed that FXII and HK compete for 220 binding sites per DXS chain. FXII and its complexes occupy one binding site, whereas HK and its complexes occupy one and a half binding sites.

#### 3.1.2. Low concentration DXS

**Fig 4:**
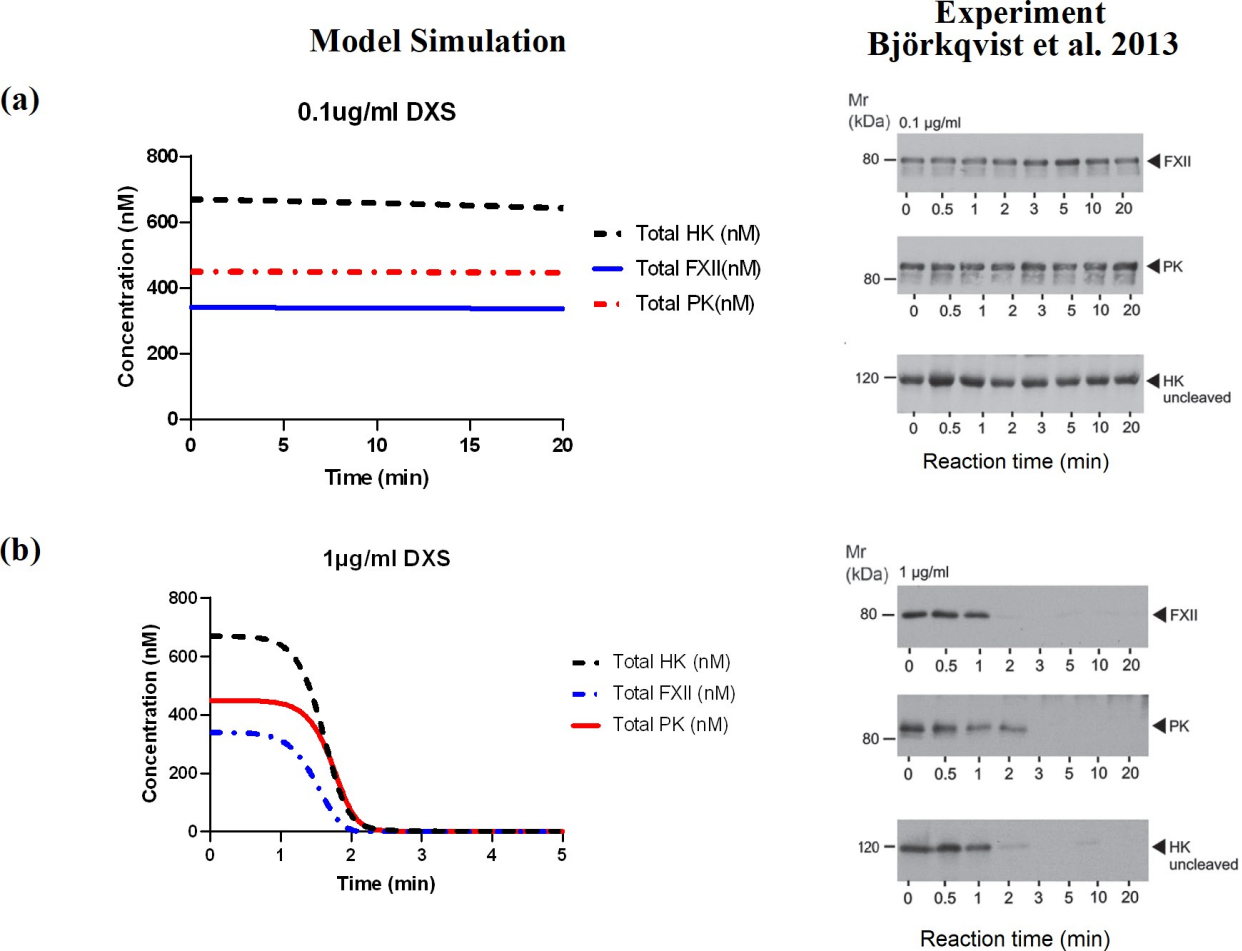
Comparison of simulations of the activation of FXII, PK and cleavage of HK with the experimental data from Björkqvist et al [30]. a) [DXS] = 0.1 μg/ml, b) [DXS] = 1 μg/ml.

Björkqvist et al. [29] incubated platelet-poor plasma with 0.1 μg/ml and 1 μg/ml DXS and analysed the activation of the KKS proteins by monitoring the disappearance of the zymogens via Western blotting. For more detailed information about the experimental procedures, the reader is referred to the paper by Björkqvist et al. [29]. In these experiments, the rates of surface-mediated reactions were limited due to a scarcity of DXS binding sites. As shown in Fig 4, we simulated the rate and extent of activation of FXII and PK, as well as cleavage of HK in the presence of 0.1 μg/ml and 1 μg/ml DXS and compared the results with the experimental data from Bjorkqvist et al. [29]. The model simulations are in good agreement with the experimental data and show complete activation of FXII and PK as well as complete cleavage of HK by 1 μg/ml DXS after 3 minutes. Furthermore, again reflecting the findings of Björkqvist et al. [29], no activation of FXII and PK, and minimal cleavage of HK in the presence of 0.1 μg/ml DXS was predicted by the model. It should be noted that the exact concentrations of species at different time points cannot be determined in Western blot experiments. Therefore, we only considered the time points at which FXII, PK, or HK were completely consumed for model calibration.

#### 3.1.3. High concentration DXS

**Fig 5:**
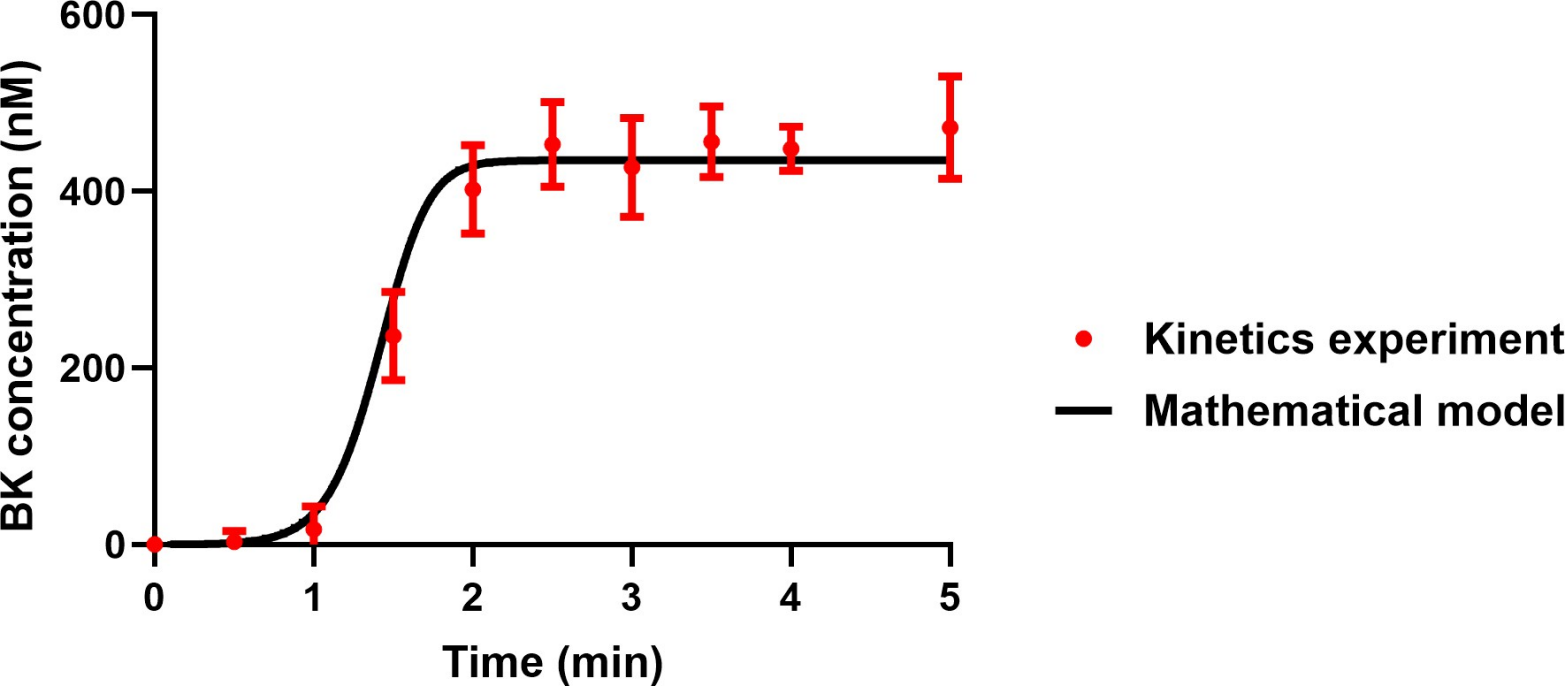
The level of BK generated by addition of 100 μg/mL DXS to plasma; comparison between in vitro data and model output. In the LC-MS/MS assay, the BK level was tested at 10 different time points. The error bars represent the standard deviation of the BK levels in four tested plasma samples.

Using the LC-MS/MS assay described in Section 2.3, we measured the kinetics of BK formation in four plasma samples from healthy donors. In parallel, we simulated plasma activation at a DXS concentration of 100 μg/mL, applying the same model parameters used to generate the simulations presented in Section 3.2.1. However, we excluded the BK degradation reaction due to the presence of the protease inhibitor mixture in the assay. We set the DXS concentration to 200 nM in the forward rate of surface reactions, scaled up the number of binding sites in proportion to the DXS concentration, and considered the plasma dilution factor. The maximum concentration of BK in the model was 435 nM, corresponding to the cleavage of all HK molecules in the system, with over 90% of BK being released within the first two minutes after the addition of DXS. Similarly, in all plasma samples tested in the LC-MS/MS assay, more than 90% of BK was released within the first two minutes; however, the maximum measured BK levels varied from 419 to 506 nM. It is important to note that there is a high degree of uncertainty regarding the initial concentrations of various zymogens, such as FXII, PK and HK in plasma, due to variability from one donor to another. For example, the reported initial concentration of HK ranges from 70 μg/mL to 175 μg/mL [35,46], leading to similar variations in the total BK levels (see S3 Fig). Therefore, for model validation, we compared the BK generation level in the model with the average BK level from four tested plasma samples. As depicted in Fig 5, the model outputs closely aligned with the experimental data, demonstrating the model’s capacity to accurately reflect the impact of high DXS concentration on the apparent rate of surface reactions.

### 3.2. BK generation in HAE plasma is faster and higher than in healthy plasma

**Fig 6:**
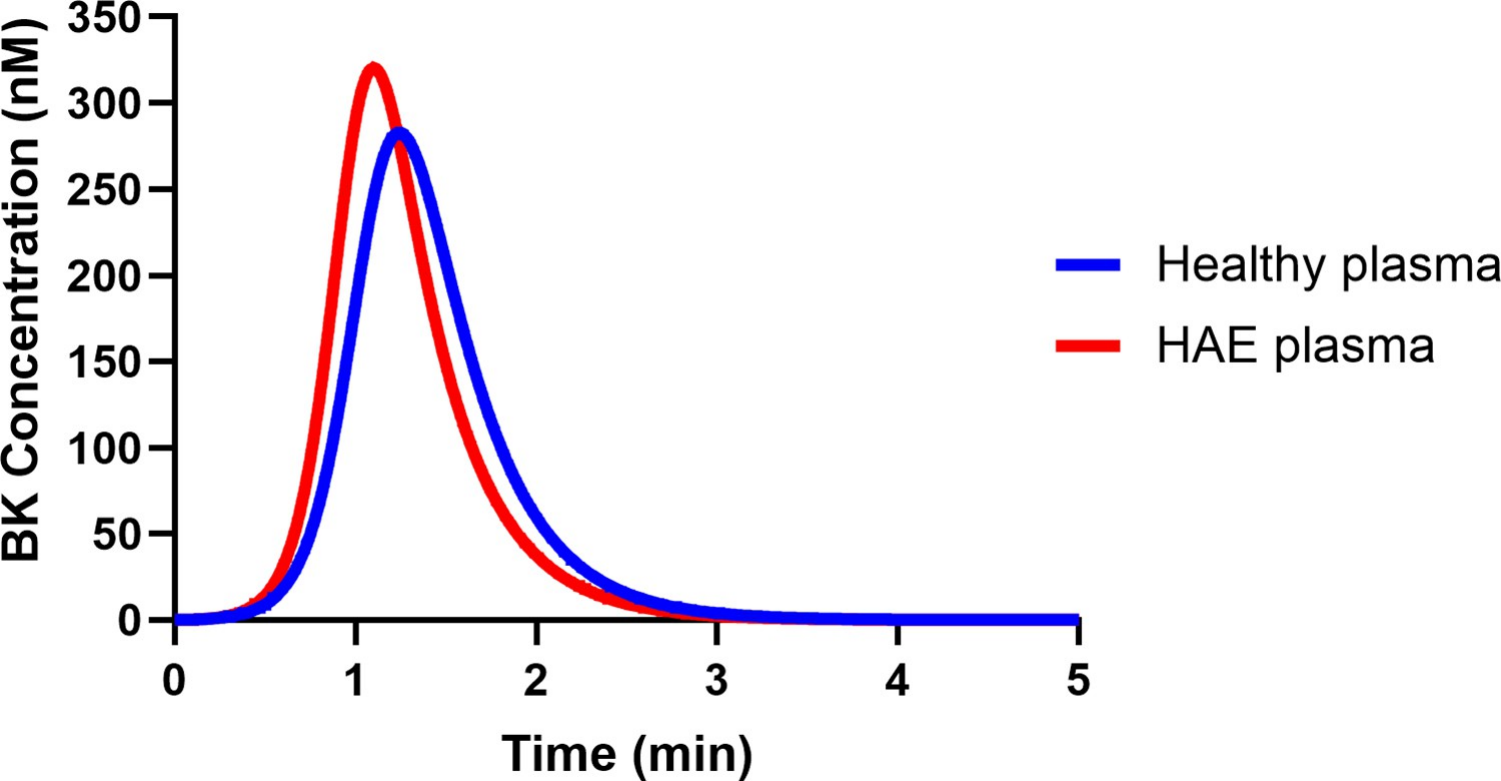
Model simulation comparison of BK generation in healthy and HAE plasma in the presence of 100 μg/ml DXS.

We simulated BK generation in HAE plasma in the presence of 100 μg/mL DXS, without any plasma dilution, and assumed that the concentration of C1inh in HAE plasma is 10% of that in healthy plasma [47]. The results indicated that the deficiency of C1inh due to HAE not only resulted in increased BK levels but also an accelerated rate of BK formation as illustrated in Fig 6. The simulations indicated the peak concentration of BK in HAE plasma (320.2 nM) was about 1.13 times higher than the peak concentration of BK in healthy plasma (282.7 nM). The time to peak BK concentration in healthy and HAE plasma was respectively, 75 and 66 seconds.

### 3.3. CSL312 proves more efficacious than ecallantide and C1inh in inhibiting BK generation

**Fig 7:**
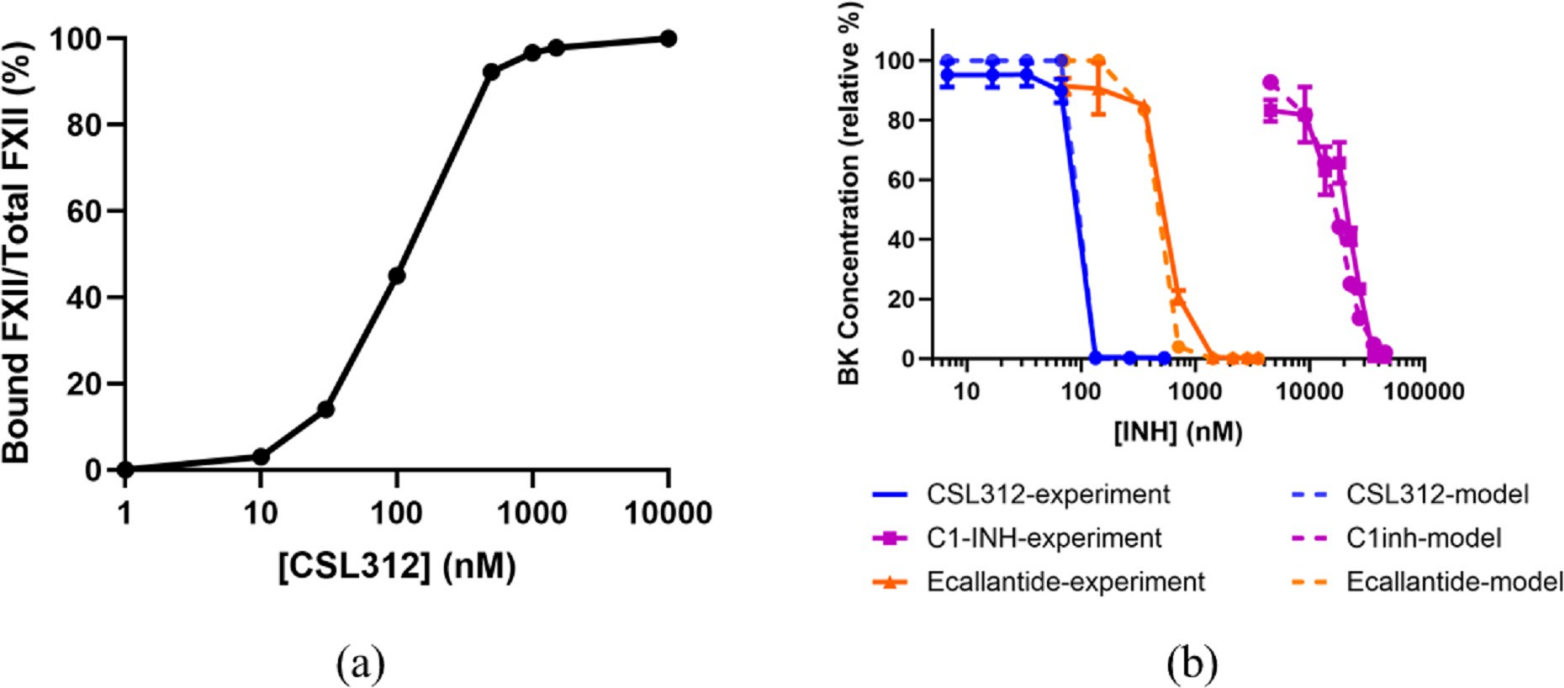
Inhibition of BK generation via different KKS inhibitors. a) Simulated data showing the proportion of CSL312-FXII complex to total FXII in plasma equilibrated with different concentrations of CSL312, b) normalised BK concentrations generated by DXS-mediated activation of healthy pooled plasma titrated with various KKS inhibitors, comparison of *in vitro* data with model output. Experimental data was adapted from Cao et al. [8].

The effect of CSL312, ecallantide, and C1inh on the normalised level of BK, five minutes after the addition of DXS, was modelled and the results were compared with published experimental data [8]. The affinities of CSL312 to both FXII and FXIIa, as well as the affinity of ecallantide to PKa, were extracted from the literature, and are listed in S3 Table. As CSL312 binds to both the zymogen and active forms of FXII, simulation of the effect of CSL312 on BK formation was performed in two steps in order to replicate the preincubation of antibody with plasma as per the experimental method. First the binding of CSL312 to FXII was modelled to steady-state, shown in Fig 7A, and then FXII activation due to the subsequent addition of DXS to the mixture was simulated. The simulations are in good agreement with the published experimental data (Fig 7B), and indicate that CSL312 has a higher potency compared to ecallantide and C1inh for the inhibition of BK.

## 4. Discussion

### 4.1. Development of the mechanistic model of KKS

In this study, we developed and validated a mechanistic model for the KKS using multiple published data sets, addressing challenges such as the high degree of uncertainty in kinetic parameters of biochemical reactions and the dependency of surface-mediated kinetic parameters on (negatively-charged) surface concentration. We focused on the core components of the KKS as typically delineated in the literature, with the aim of providing a clear and concise model structure. While we recognize that all biological systems are interconnected, our review of the literature did not reveal any significant feedback from downstream interactions or other biological pathways such as the coagulation and complement cascades that would materially impact the outcomes predicted by our model. We focused on DXS as a well-established trigger of the contact activation system, distinguished between surface and volume reactions in the KKS, and optimised parameters that best fit several experimental data sets.

We identified reactions with kinetics that depend strongly on the concentration of surface-bound species including FXII self-activation, the activation of surface-bound FXII by the PKa-HK complex, the conversion of surface-bound αFXIIa to βFXIIa, the activation of the HK-PK complex by surface-bound αFXIIa, and PK self-activation at the surface. To capture the impact of binding site concentration on these surface-mediated reaction rates, we formulated mass-action equations and substantiated their accuracy by aligning our model predictions with empirical data from BK kinetics experiments performed at varying DXS concentrations.

The model accounts for the effect of DXS binding site concentration on FXII activation and includes the surface binding of HK and its complexes. PK/PKa binds to HK, and the resulting complex competes with FXII for surface sites. Consequently, the rate of FXII activation by PKa-HK on the surface depends on the concentration of surface sites and exhibits a bell-shaped curve dependency, similar to the FXII self-activation reaction. Furthermore, the activation of PK by αFXIIa in the presence of HK also involves competition for surface sites. However, it has been shown that, unlike FXII activation, the rate of PK activation by αFXIIa plateaus when the surface sites are in excess [48], and does not vary with HK concentration. This is likely due to the rapid conversion of surface-bound αFXIIa to the fluid-phase βFXIIa by PKa, which can then activate PK independently of the surface site concentration.

Our findings revealed that there is a threshold concentration of DXS required to activate the system. Below this threshold concentration, very little FXII is activated and therefore the inhibitors are able to maintain the system in a quiescent state. As shown in S5 Fig, when the concentration of DXS surpasses this threshold, the system rapidly becomes activated, leading to the cleavage of all HK molecules and the subsequent release of BK. The activation rates of PK-HK by αFXIIa and FXII by PKa-HK at the surface are much faster than in the volume, so increasing the number of binding sites enhances the rate of BK generation. However, further increases in surface site concentration would lead to a decrease in the average concentration of surface-bound species, resulting in fewer reaction events and a reduced rate of BK generation. S6 Fig depicts the effect of various DXS concentrations (0.1, 1, 10, 100, 1000, and 10000 μg/ml) on BK generation. It demonstrates that BK generation occurs more rapidly at a concentration of 10 μg/ml DXS compared to both lower (0.1 and 1 μg/ml) and higher (100, 1000, and 10000 μg/ml) concentrations.

### 4.2. Mechanism of FXII activation

Using mathematical modelling, we tested two hypotheses regarding the initiation of contact activation. One hypothesis posits that a trace amount of FXIIa, already present in plasma, is required for further FXII activation, while the other suggests that FXII activation occurs due to conformational changes following adsorption onto negatively-charged surfaces. Our simulations indicated that FXII autoactivation leads to a gradual but sustained activation of FXII, particularly pronounced when FXII is incubated with high DXS concentrations for 30 minutes, which contradicts experimental observations. Consequently, the second hypothesis; that a trace amount of FXIIa is required for further FXII activation, appears more plausible. Nevertheless, the exact mechanism behind the initial activation of FXII in a static closed plasma system is not critical, as the FXII-PKa feedback loop quickly becomes the predominant pathway for FXII activation.

### 4.3. Comparative analysis of different KKS inhibitors

Published data comparing the potency of CSL312, ecallantide and C1inh in the regulation of the KKS showed that CSL312 was the most potent BK inhibitor [8]. We used our mechanistic model to simulate these different treatments to gain insight into their mechanism of action, as outlined below.

Although C1inh plays a key role in inhibiting FXIIa and PKa, its affinity for these enzymes is relatively low. As a result, very high concentrations of C1inh are required to completely block BK generation, making it less effective than CSL312 or ecallantide. Interestingly, while C1inh can bind to both PKa and FXIIa, its deficiency primarily affects FXII activation, as, *in vivo*, it is responsible for inhibiting 93% of FXIIa [49]. Alpha-2-macroglobulin can partially compensate for the lack of C1inh inhibition of PKa, as C1inh accounts for only 52% of PKa inhibition [47]. Consequently, C1inh deficiency in HAE patients has a greater effect on FXII activation than that of PK activation.

CSL312 is a high-affinity blocking antibody that targets both FXII zymogen and its active enzymatic forms, αFXIIa and βFXIIa. Each CSL312 antibody can bind to two FXII/FXIIa molecules and is capable of preventing the activation of FXII to αFXIIa as well as inhibiting the catalytic activities of both αFXIIa and βFXIIa. By binding the FXII zymogen prior to the addition of DXS to plasma, CSL312 blocks a significant proportion of FXII from activation as illustrated in Fig 7A. Understanding the mechanism that triggers FXII activation is crucial for accurately describing the behaviour of CSL312, particularly under conditions of blood flow. Our findings indicated that a trace amount of FXIIa is essential for the further activation of FXII. Given that CSL312 exhibits high affinity for FXIIa and can completely inhibit low background levels of FXIIa *in vivo*, it effectively blocks the generation of bradykinin.

The catalytic efficacy of a reaction is a measure of how effectively an enzyme or catalyst facilitates a chemical reaction, and is mathematically expressed as k_cat_/K_M_. A higher catalytic efficiency indicates that the enzyme is more effective at converting substrate into product. The catalytic efficiency for the activation of PK by FXIIa is higher than that of FXII activation by PKa, resulting in a greater rate of PK activation. Therefore, to effectively impede the reciprocal FXII-PK activation loop, and thereby reduce KKS activity, inhibition of FXIIa is a more effective approach than inhibition of PKa. This increased rate of PK activation by FXIIa is further amplified by virtue of the surface-binding behaviours of the two proteins. Both surface-bound αFXIIa and fluid-phase βFXIIa activate PK, regardless of the location of PK. However, FXII activation predominantly occurs at the surface and the rate of fluid-phase FXII activation by PKa is negligible. To demonstrate the advantage of targeting FXIIa over PKa, we compared the efficacy of CSL312 with a hypothetical antibody that can bind to both PK and PKa with the same affinity as that of CSL312 to FXII/FXIIa, as shown in S7 Fig. The results showed that CSL312 is a significantly more potent BK inhibitor than the hypothetical PK/PKa antibody.

### 4.4. Limitations

In this study, we have developed a mechanistic model of the plasma KKS under *in vitro* conditions and in the absence of endothelial cells or any tissue compartment. It should be noted that the rate of FXII activation *in vivo* may vary under different conditions and depending on the negatively charged activating surface. This variability prompted us to focus solely on DXS, a well-known potent activator of the contact pathway. DXS has been extensively used to initiate the KKS, enabling us to find reliable experimental results in the literature for model validation. In our model, the equations that describe the interactions of surface-bound molecules assume that the reaction rate is proportional to the reactants’ surface densities. This means that these surface-bound molecules can freely diffuse over the surface, facilitating interactions between molecules that are not initially adjacent, thereby increasing the likelihood of reaction events. However, if molecules cannot diffuse across the surface and remain anchored at their binding sites, the model’s assumptions might not hold. Under such conditions, reaction rates would depend more on the initial spatial distribution of the molecules on the surface rather than on their overall density. This aspect becomes particularly critical in the presence of a heterogeneous distribution of molecules on the surface. Consequently, the probability of a reaction would be higher for molecules that are in close proximity to one another. However, describing such a phenomenon would require the use of partial differential equations, which was beyond the scope of this study.

The model relies on numerous kinetic and stoichiometric parameters. Despite employing optimisation, the limited number of datasets means these optimised parameters may not be necessarily unique. For instance, differences may exist between the inactivation rates of free versus surface-bound enzymes by various inhibitors in the system, with surface-bound enzymes potentially being completely or partially protected from inhibition. However, without additional data to **®**ort the introduction of different rates between bound and free enzymes, we are unable to validate this hypothesis. Additionally, there is conflicting evidence about the role of HK in partially protecting PKa from inhibition by C1inh, α2M, and AT, as indicated in several studies [31,37–39,50,51]. Although our aim was to identify optimised values for all reactions through model calibration, the results from sensitivity analysis suggested that kinetic rates related to the inhibitory activities of AT and α2AP, show low sensitivity. However, the fact that the simulations correspond with experimental outcomes across various DXS concentrations and under different drug interactions, each with a unique mechanism of action, lends greater confidence that the reaction constants used are robust.

Looking ahead, several steps are recommended to enhance the utility of the model and to ascertain its capacity to contribute effectively to medical advancements and patient outcomes. First, although this model has been developed for a closed, well-mixed system, the effects of blood flow and diffusion might be crucial in predicting the efficacy of different targets in the HAE treatment. HAE attacks are often considered localized phenomena, and incorporating flow and diffusion could significantly improve model accuracy by realistically simulating the supply of fresh zymogens to the region of HAE attack and the washout of products. Secondly, further investigation is required to understand the mechanisms that trigger KKS activation in HAE patients, especially those with a normal level of C1 inhibitor but mutations in the FXII or plasminogen genes. Lastly, prospective validation studies need to be conducted to confirm the model’s predictions *in vivo*, covering a diverse range of clinical scenarios.

### 4.5. Conclusion

The mechanistic model, representing the KKS, provides significant advancement in our understanding of this intricate biological pathway. The meticulous fitting of the model against published experimental data has enabled an accurate depiction of the KKS dynamics, particularly enhancing our understanding of the impact of initial FXII activation triggers, as well as the sensitivity of the contact system to the concentration of DXS surface binding sites. Furthermore, the rigorous validation of the model against independent data sets has established its robustness and reliability in predictive capacities. The model unravels the mechanism of action of different KKS inhibitors under *in vitro* conditions, shedding light on the reasons behind the higher efficacy of CSL312 compared to ecallantide and C1inh. Such a comprehensive and validated mechanistic model can be a predictive tool in guiding the development of new therapeutics and refining existing treatments, ultimately aiming to improve patient outcomes in conditions influenced by KKS dysregulation.

## Supporting information

S1 Text Description of the mechanism of FXII activation. (PDF)

S1 Table Initial concentrations of binding sites (S) and different zymogens in the computational model [16]. It was assumed that FXII and its complexes occupy one binding site, while HK and its complexes occupy one and a half binding sites.
(PDF)

S2 Table The ordinary difference equations for the plasma kallikrein-kinin system model. Surface-bound species are indicated by subscript ‘s’, and solution-phase reactions are indicated by subscript ‘v’. Kinetic parameters ka, kd, and kcat for different reactions are reported in the Table 1.
(PDF)

S3 Table List of biochemical reactions for CSL312 and ecallantide (DX88). Surface-bound species indicated by a subscript ‘s’. It is important to note that the binding affinity of CSL312 to FXII zymogen, as measured in-house via surface plasmon resonance (SPR), may not be accurate. This uncertainty primarily arises from challenges in obtaining pure FXII zymogen. The presence of small amounts of FXIIa, known to have a significantly higher affinity for CSL312, may result in an overestimation of the zymogen binding affinity. To assess the impact of this uncertainty on BK inhibition, we conducted simulations using an order of magnitude lower affinity of CSL312 for the FXII zymogen. The results indicated a negligible difference in BK generation levels.
(PDF)

S1 Fig The comprehensive scheme of the plasma KKS network model. FXII: factor FXII, αFXIIa: two-chain activated factor FXII, βFXIIa: single-chain activated FXII, FXI: factor FXI, PK: prekallikrein, PKa: kallikrein, HK: high molecular weight kininogen, cHK: cleaved high molecular weight kininogen, BK: bradykinin, α2M: α2-macroglobulin, C1inh: C1 esterase inhibitor. Surface-bound species are indicated by subscript ‘s’, and solution-phase reactions are indicated by subscript ‘v’. Reaction numbers correspond to those in Table 1.
(TIF)

S2 Fig Effect of uncertainty in the activation rate of FXII by kallikrein on the BK level. The activation rate of r37 was varied from 10% to 1000% of the baseline value, and the corresponding variations in BK levels were determined.
(TIF)

S3 Fig Sensitivity of model output to the initial concentrations of species. Each initial concentration was varied from 10% to 1000% of the baseline value, and the effects on the time required to generate 20 nM of BK, as well as the maximum BK level, were determined. The sensitivity index factor was calculated by dividing the variations in the time to generate 20 nM of BK or in the maximum BK level by their corresponding baseline values.
(TIF)

S4 Fig Sensitivity of model output to kinetic parameters. Each kinetic parameter was individually varied from 10% to 1000% of the baseline value, and the effects on the time required to generate 20 nM of BK, as well as the maximum BK level, were determined. The sensitivity index factor was calculated by dividing the variations in the time to generate 20 nM of BK or in the maximum BK level by their corresponding baseline values. This factor was depicted for the ten most sensitive parameters.
(TIF)

S5 Fig The impact of low concentrations of DXS (below 1μg/ml) on the BK generation. A stability zone exists at low concentrations of DXS, with no significant BK generation occurring. When the concentration of DXS exceeds a certain threshold, the steady state no longer exists, and complete cleavage of HK is observed.
(TIF)

S6 Fig The effect of various DXS concentrations (above 1μg/ml) on the rate of BK generation. The modelling results are consistent with our experimental data showing that at 3 minutes after the addition of 1, 10, and 100 μg/ml DXS, the level of BK is the same and maximal.
(TIF)

S7 Fig Comparison of the potency of 150nM CSL312 and a hypothetical antibody that binds to PK and PKa with the same affinity that CSL312 binds to FXII and FXIIa. The concentration of DXS is 100μg/ml and the plasma dilution factor was 0.65.
(TIF)
