# Supplementary material for: A mechanistic model of in vitro plasma activation to evaluate therapeutic kallikrein-kinin system inhibitors
Source: PLoS Comput Biol. 2024 Nov 4;20(11):e1012552. doi: 10.1371/journal.pcbi.1012552 (PMC11563367; doi:10.1371/journal.pcbi.1012552)
Supplement: S1 Text — (PDF) [file pcbi.1012552.s001.pdf]

Supporting information for “A mechanistic model of in vitro plasma activation to evaluate therapeutic kallikrein-kinin system inhibitors”

Description of the mechanism of FXII activation

The activation of FXII can be described through five reactions as follows:

Initial binding of FXII to the surface:

$$FXII_v + S_s \underset{k_{d,1}}{\overset{k_{a,1}}{\Longleftrightarrow}} (FXII{:}S)_s \quad \text{(s1)}$$

Binding of activated FXII to the surface:

$$\alpha FXIIa_v + S_s \underset{k_{d,2}}{\overset{k_{a,2}}{\Longleftrightarrow}} (\alpha FXIIa{:}S)_s \quad \text{(s2)}$$

FXII auto-activation:

$$FXII_s \overset{k_3}{\Longrightarrow} \alpha FXIIa_s \quad \text{(s3)}$$

FXII self-activation:

$$(FXII{:}S)_s + (\alpha FXIIa{:}S)_s \underset{k_{d,4}}{\overset{k_{a,4}}{\Longleftrightarrow}} (FXII{:}S{:}\alpha FXIIa{:}S)_s \overset{k_4}{\Longrightarrow} 2(\alpha FXIIa{:}S)_s \quad \text{(s4)}$$

Cleavage of αFXIIa by other αFXIIa and generation of βFXIIa:

$$(\alpha FXII{:}S)_s + (\alpha FXIIa{:}S)_s \underset{k_{d,5}}{\overset{k_{a,5}}{\Longleftrightarrow}} (\alpha FXII{:}S{:}\alpha FXIIa{:}S)_s \overset{k_5}{\Longrightarrow} (\alpha FXIIa{:}S)_s + \beta FXIIa_v + S_s \quad \text{(s5)}$$

The rate expression for the reactions 1 to 5 would be:

$$R_{1s} = k_{a\_1}\,[FXII_v][S_s] - k_{d\_1}\,[(FXII{:}S)_s] \quad \text{(s6)}$$

$$R_{2s} = k_{a\_2}\,[\alpha FXIIa_v][S_s] - k_{d\_2}\,[(\alpha FXIIa{:}S)_s] \quad \text{(s7)}$$

$$R_{3s} = k_{a\_3}\,[FXII_s] \quad \text{(s8)}$$

$$R_{4s,i} = k_{a\_4}[(FXII{:}S)s\,][\alpha FXIIa{:}S)s] - k_{d\_4}[(FXII{:}S - \alpha FXIIa{:}S)s] \quad \text{(s9)}$$

$$R_{4s,ii} = k_{cat\_4}[(FXII{:}S - \alpha FXIIa{:}S)s] \quad \text{(s10)}$$

$$R_{5s,i} = k_{a\_5}[(\alpha FXIIa{:}S)s\,][\alpha FXIIa{:}S)s] - k_{d\_5}[(\alpha FXIIa{:}S - \alpha FXIIa{:}S)s] \quad \text{(s11)}$$

$$R_{5s,ii} = k_{cat\_5}[(\alpha FXIIa{:}S - \alpha FXIIa{:}S)s] \tag{s12}$$

The rates of the reactions s6-s12 all have units of mol. m^{-2}. s^{-1} and are therefore the reaction rates per surface of DXS molecule (shown by R_s). Note that the (surface based) rate constants in reactions 1-5 are assumed to be the fundamental mass-action rate constants that do not vary with DXS concentration. However, the major consequence of measuring FXII self-activation in a volume-based reaction vessel (as is the norm), is that the reported volume-based kinetic parameters might contain an embedded dependence on the activator (surface) concentration used in the experiment, and so may not necessarily be constant.

Assuming that the system is well mixed, there are two sensible reference frames relative to which the concentrations of surface-bound species may be reported. Taking the DXS chains as the reference frame, the surface concentration s_{is} (mol/m^2) of species i is the total amount of surface-bound i in the reaction vessel (n_{is}) divided by the total surface area of all DXS chains in the reaction vessel (A_{DXS}), that is:

$$s_{is} = \frac{n_{is}}{A_{DXS}} \tag{s13}$$

(Because the system is homogeneous, the time-averaged surface concentration on each DXS chain will be the same.) Taking the reaction vessel as the reference frame, the volume concentration c_{is} (mol/m^3) of species i is the total amount of surface-bound i in the reaction vessel (n_{is}) divided by the volume of the reaction vessel (V_{tot}), that is:

$$c_{is} = \frac{n_{is}}{V_{tot}} \tag{s14}$$

therefore, the total amount of any DXS-bound species, i, will be:

$$n_{is} = c_{is} \times V_{tot} = s_{is} \times A_{DXS} \tag{s15}$$

Rearranging this results in:

$$\frac{c_{is}}{s_{is}} = \frac{A_{DXS}}{V_{tot}} = \sigma_{DXS} \tag{s16}$$

where σ_{DXS} is the specific area of the DXS molecules. Similarly, the total reaction rate in the vessel for any of the surface reactions can be written as:

$$\frac{R_v}{R_s} = \frac{A_{DXS}}{V_{tot}} = \sigma_{DXS} \tag{s17}$$

where R_s is an individual surface rate (as per reactions 1-5, in mol/(m^2.s)) and R_v is the equivalent volume-based rate (in mol/(m^3.s)). Equations s16 and s17 allow the (possibly

changing) volumetric rate constants to be derived from the (DXS concentration independent) surface-based rate constants.

To illustrate, we now perform this process for some typical surface reactions. The (surface) rate expression for reaction (1) was shown in equation 5, where $k_{a,1}$ and $k_{d,1}$ are assumed to be independent of DXS concentration, and we have adopted the standard $[i]$ notation for volume concentration c_i. Substituting in expressions for $R_{1,s}$, s_S and s_{FXII_s} based on equations s16 and s17 gives:

$$\frac{R_{1v}}{\sigma_{DXS}} = k_{a\_1}\,[FXII]\frac{[S_s]}{\sigma_{DXS}} - k_{d\_1}\,\frac{[(FXII - S)_s]}{\sigma_{DXS}} \quad (s18)$$

which simplifies to:

$$R_{1v} = k_{a\_1}\,[FXII][S_s] - k_{d\_1}\,[(FXII - S)_s] \quad (s19)$$

so, there is no dependence of volume-based rate constants R_{1v} in reaction (1) on DXS concentration. Conducting the same procedure for reactions 2-4 we find that the volume-based kinetic constants for reactions 2 and 3 also have no dependence on DXS concentration. However, when two surface-bound species interact, as is the case in the reversible part of reaction 4 (termed 4i), we find:

$$R_{4s,i} = \frac{R_{4v,i}}{\sigma_{DXS}} = k_{a\_4}\frac{[(FXII{:}S)_s]}{\sigma_{DXS}}\frac{[(\alpha FXIIa{:}S)_s]}{\sigma_{DXS}} - k_{d\_4}\frac{[(FXII{:}S - \alpha FXIIa{:}S)_s]}{\sigma_{DXS}} \quad (s20)$$

which after simplification becomes:

$$R_{4v,i} = \frac{k_{a\_4}}{\sigma_{DXS}}\,[(FXII{:}S)_s][(\alpha FXIIa{:}S)_s] - k_{d\_4}\,[(FXII{:}S - \alpha FXIIa{:}S)_s] \quad (s21)$$

Hence, when expressed on a volume basis the effective forward rate constant for this reaction varies with σ_{DXS}, which itself depends on the molecular weight (Mw) of the DXS, via the area of each DXS chain, as well as the concentration of DXS chains. To convert this to an expression that is universally applicable to DXS of any molecular weight, we note that:

$$\sigma_{DXS} = \frac{A_{DXS}}{V_{Tot}} = [DXS]A_{1DXS}N_A \quad (s22)$$

where A_{1DXS} is the area of a single DXS chain of the specific Mw of DXS being used, and N_A is Avogadro's constant. Replacing equation s22 into equation s21 results in:

$$R_{4v,i} = \frac{k_{a\_4}}{[DXS]A_{1DXS}N_A}\,[(FXII{:}S)_s][(\alpha FXIIa{:}S)_s] - k_{d\_4}\,[(FXII{:}S - \alpha FXIIa{:}S)_s] \quad (s23)$$

In which, $k_{a\_4} \times A_{1DXS}^{-1} \times N_A^{-1}$ is constant and can be replaced by $\widetilde{k_{a\_4}}$.

$$R_{4v,i} = \frac{\widetilde{k_{a\_4}}}{[DXS]} [(FXII:S)_s][(\alpha FXIIa:S)_s] - k_{d\_4} [(FXII:S - \alpha FXIIa:S)_s] \qquad \text{(s24)}$$

Similarly, for other reactions where two surface-bound species interact, the same procedure can be applied.
