# Supplementary material for: A mechanistic model of in vitro plasma activation to evaluate therapeutic kallikrein-kinin system inhibitors
Source: PLoS Comput Biol. 2024 Nov 4;20(11):e1012552. doi: 10.1371/journal.pcbi.1012552 (PMC11563367; doi:10.1371/journal.pcbi.1012552)
Supplement: S1 Table — It was assumed that FXII and its complexes occupy one binding site, while HK and its complexes occupy one and a half binding sites. (PDF) [file pcbi.1012552.s002.pdf]

| Species | Initial concentration (nM) | Molecular weight (kDa) |
|---|---|---|
| S | 220 x [DXS] | 500 |
| FXII | 340 | 80 |
| PK | 450 | 88 |
| PKa | 0 | 88 |
| HK | 670 | 120 |
| cHK | 0 | 110 |
| BK | 0 | 1.06 |
| FXI | 31 | 160 |
| α2M | 1750 | 718 |
| C1inh | 2500 | 110 |
| AT | 2600 | 58 |
| α2AP | 1000 | 70 |
