# Supplementary material for: A mechanistic model of in vitro plasma activation to evaluate therapeutic kallikrein-kinin system inhibitors
Source: PLoS Comput Biol. 2024 Nov 4;20(11):e1012552. doi: 10.1371/journal.pcbi.1012552 (PMC11563367; doi:10.1371/journal.pcbi.1012552)
Supplement: S2 Table — Surface-bound species are indicated by subscript ‘s’, and solution-phase reactions are indicated by subscript ‘v’. Kinetic parameters ka, kd, and kcat for different reactions are reported in the Table 1. (PDF) [file pcbi.1012552.s003.pdf]

1	d([(PKa-cHK-S)_s])/dt = 1/(((ka_15*PKa_v*[(cHK-S)_s])-(kd_15*[(PKa-cHK-S)_s])) + ((ka_27*[(PKa-cHK)_v]*[1.5*S_s])-(kd_27*[(PKa-cHK-S)_s])) + ((kcat_30*[(PK-cHK-S-αFXIIa-S)_s])) - ((ka_38*[(FXII-S)_s]*[(PKa-cHK-S)_s])-(kd_38*[(FXII-PKa-cHK-S)_s])) + ((kcat_38*[(FXII-PKa-cHK-S)_s])) - ((ka_40*[(αFXIIa-S)_s]*[(PKa-cHK-S)_s])-(kd_40*[(αFXIIa-S-PKa-cHK-S)_s])) + ((kcat_40*[(αFXIIa-S-PKa-cHK-S)_s])) + ((ka_42*[(PK-cHK-S)_s]*[(PKa-HK-S)_s])) + ((ka_44*[(PK-cHK-S)$_s$]*[(PKa-cHK-S)_s])) + ((kcat_45*[(PKa-HK-S)_s])) - ((ka_66*[(PKa-cHK-S)_s]*C1inh_v)) - ((ka_72*[(PKa-cHK-S)_s]*AT_v)))
2	d(AT_v)/dt = 1/(-((ka_57*[αFXIIa_v]*AT_v)) - ((ka_58*[(αFXIIa-S)_s]*AT_v)) - ((ka_61*[βFXIIa_v]*AT_v)) - ((ka_68*PKa_v*AT_v)) - ((ka_69*[(PKa-HK)_v]*AT_v)) - ((ka_70*[(PKa-HK-S)_s]*AT_v)) - ((ka_71*[(PKa-cHK)_v]*AT_v)) - ((ka_72*[(PKa-cHK-S)_s]*AT_v)))
3	d([(PKa-cHK-S- AT)_s])/dt = 1/(((ka_72*[(PKa-cHK-S)_s]*AT_v)))
4	d([(PKa-cHK)_v])/dt = 1/(((ka_14*PKa_v*cHK_v)-(kd_14*[(PKa-cHK)_v])) - ((ka_27*[(PKa-cHK)_v]*[1.5*S_s])-(kd_27*[(PKa-cHK-S)_s])) + ((kcat_33*[(PK-cHK-βFXIIa)_v])) + ((kcat_48*[(PKa-HK)_v])) - ((ka_64*[(PKa-cHK)_v]*C1inh_v)) - ((ka_71*[(PKa-cHK)_v]*AT_v)))
5	d([(PKa-cHK-AT)_v])/dt = 1/(((ka_71*[(PKa-cHK)_v]*AT_v)))
6	d([(PKa-HK-S)_s])/dt = 1/(((ka_26*[(PKa-HK)_v]*[1.5*S_s])-(kd_26*[(PKa-HK-S)_s])) + ((kcat_29*[(PK-HK-S-αFXIIa-S)_s])) - ((ka_37*[(FXII-S)_s]*[(PKa-HK-S)_s])-(kd_37*[(FXII-PKa-HK-S)_s])) + ((kcat_37*[(FXII-PKa-HK-S)_s])) - ((ka_39*[(αFXIIa-S)_s]*[(PKa-HK-S)_s])-(kd_39*[(αFXIIa-S-PKa-HK-S)_s])) + ((kcat_39*[(αFXIIa-S-PKa-HK-S)_s])) + ((ka_41*[(PK-HK-S)_s]*[(PKa-HK-S)_s])) + ((ka_43*[(PK-HK-S)_s]*[(PKa-cHK-S)_s])) + ((ka_45*PKa_v*[(HK-S)_s])-(kd_45*[(PKa-HK-S)_s])) - ((kcat_45*[(PKa-HK-S)_s])) - ((ka_65*[(PKa-HK-S)_s]*C1inh_v)) - ((ka_70*[(PKa-HK-S)_s]*AT_v)))
7	d([(PKa-HK-S-AT)_s])/dt = 1/(((ka_70*[(PKa-HK-S)_s]*AT_v)))
8	d([(PKa-HK)_v])/dt = 1/(-((ka_26*[(PKa-HK)_v]*[1.5*S_s])-(kd_26*[(PKa-HK-S)_s])) + ((kcat_32*[(PK-HK-βFXIIa)_v])) - ((ka_35*FXII_v*[(PKa-HK)_v])-(kd_35*[(FXII-PKa-HK)_v])) + ((kcat_35*[(FXII-PKa-HK)_v])) - ((ka_36*FXII_v*[(PKa-HK)_v])-(kd_36*[(FXII-PKa-HK)_v])) + ((kcat_36*[(FXII-PKa-HK)_v])) + ((ka_48*PKa_v*HK_v)-(kd_48*[(PKa-HK)_v])) - ((kcat_48*[(PKa-HK)_v])) - ((ka_63*[(PKa-HK)_v]*C1inh_v)) - ((ka_69*[(PKa-HK)_v]*AT_v)))
9	d([(PKa-HK-AT)_v])/dt = 1/(((ka_69*[(PKa-HK)_v]*AT_v)))
10	d(PKa_v)/dt = 1/(-((ka_14*PKa_v*cHK_v)-(kd_14*[(PKa-cHK)_v])) - ((ka_15*PKa_v*[(cHK-S)_s])-(kd_15*[(PKa-cHK-S)_s])) + ((kcat_28*[(PK-αFXIIa-S)_s])) + ((kcat_31*[(PK-βFXIIa)_v])) - ((ka_34*FXII_v*PKa_v)-(kd_34*[(FXII-PKa)_v])) + ((kcat_34*[(FXII-PKa)_v])) - ((ka_45*PKa_v*[(HK-S)_s])-(kd_45*[(PKa-HK-S)_s])) - ((ka_48*PKa_v*HK_v)-(kd_48*[(PKa-HK)_v])) - ((ka_62*PKa_v*C1inh_v)) - ((ka_67*PKa_v*[α2M_v])) - ((ka_68*PKa_v*AT_v)))
11	d([(PKa-AT)_v])/dt = 1/(-((ka_18*[(PKa-AT)_v]*cHK_v)-(kd_18*[(PKa-AT-cHK)_v])) - ((ka_19*[(PKa-AT)_v]*[(cHK-S)_s])-(kd_19*[(PKa-AT-cHK-S)_s])) - ((ka_47*[(PKa-AT)_v]*[(HK-S)_s])-(kd_47*[(PKa-AT-HK-S)_s])) - ((ka_50*[(PKa-AT)_v]*HK_v)-(kd_50*[(PKa-AT-HK)_v])) + ((ka_68*PKa_v*AT_v)))
12	d([α2M_v])/dt = 1/(-((ka_55*[αFXIIa_v]*[α2M_v])) - ((ka_56*[(αFXIIa-S)_s]*[α2M_v])) - ((ka_67*PKa_v*[α2M_v])))
13	d([PKa-α2M_v])/dt = 1/(((ka_67*PKa_v*[α2M_v])))
14	d(C1inh_v)/dt = 1/(-((ka_52*[αFXIIa_v]*C1inh_v)) - ((ka_59*[βFXIIa_v]*C1inh_v)) - ((ka_62*PKa_v*C1inh_v)) - ((ka_63*[(PKa-HK)_v]*C1inh_v)) - ((ka_64*[(PKa-cHK)_v]*C1inh_v)) - ((ka_65*[(PKa-HK-S)_s]*C1inh_v)) - ((ka_66*[(PKa-cHK-S)_s]*C1inh_v)))
15	d([(PKa-C1inh-cHK-S)_s])/dt = 1/(((ka_17*[(PKa-C1inh)_v]*[(cHK-S)_s])-(kd_17*[(PKa-C1inh-cHK-S)_s])) + ((ka_66*[(PKa-cHK-S)_s]*C1inh_v)))
16	d([(PKa-C1inh-HK-S)_s])/dt = 1/(((ka_46*[(PKa-C1inh)_v]*[(HK-S)_s])-(kd_46*[(PKa-C1inh-HK-S)_s])) + ((ka_65*[(PKa-HK-S)_s]*C1inh_v)))
17	d([(PKa-C1inh-cHK)_v])/dt = 1/(((ka_16*[(PKa-C1inh)_v]*cHK_v)-(kd_16*[(PKa-C1inh-cHK)_v])) + ((ka_64*[(PKa-cHK)_v]*C1inh_v)))
18	d([(PKa-C1inh-HK)_v])/dt = 1/(((ka_49*[(PKa-C1inh)_v]*HK_v)-(kd_49*[(PKa-C1inh-HK)_v])) + ((ka_63*[(PKa-HK)_v]*C1inh_v)))
19	d(FXII_v)/dt = 1/(-((ka_1*FXII_v*S_s)-(kd_1*[(FXII-S)_s])) - ((ka_34*FXII_v*PKa_v)-(kd_34*[(FXII-PKa)_v])) - ((ka_35*FXII_v*[(PKa-HK)_v])-(kd_35*[(FXII-PKa-HK)_v])) - ((ka_36*FXII_v*[(PKa-HK)_v])-(kd_36*[(FXII-PKa-HK)_v])))
20	d(S_s)/dt = 1/(-((ka_1*FXII_v*S_s)-(kd_1*[(FXII-S)_s])) - ((ka_2*[αFXIIa_v]*S_s)-(kd_2*[(αFXIIa-S)_s])) + ((kcat_5*[(αFXIIa-S-αFXIIa-S)_s])) - 1/(((ka_20*HK_v*[1.5*S_s])-(kd_20*[(HK-S)_s])) - ((ka_21*cHK_v*[1.5*S_s])-(kd_21*[(cHK-S)_s])) - ((ka_22*[(FXI-HK)_v]*[1.5*S_s])-(kd_22*[(FXI-

	HK-S)_s])) - ((ka_23*[(FXI-cHK)_v]*[1.5*S_s])-(kd_23*[(FXI-cHK-S)_s])) - ((ka_24*[(PK-HK)_v]*[1.5*S_s])-(kd_24*[(PK-HK-S)_s])) - ((ka_25*[(PK-cHK)_v]*[1.5*S_s])-(kd_25*[(PK-cHK-S)_s])) - ((ka_26*[(PKa-HK)_v]*[1.5*S_s])-(kd_26*[(PKa-HK-S)_s])) - ((ka_27*[(PKa-cHK)_v]*[1.5*S_s])-(kd_27*[(PKa-cHK-S)_s]))) + ((kcat_39*[(αFXIIa-S-PKa-HK-S)_s])) + ((kcat_40*[(αFXIIa-S-PKa-cHK-S)_s])))
21	d([(FXII-S)_s])/dt = 1/(((ka_1*FXII_v*S_s)-(kd_1*[(FXII-S)_s])) - ((ka_3*[(FXII-S)_s])) - ((ka_4*[(FXII-S)_s]*[(αFXIIa-S)_s])-(kd_4*[(FXII-S-αFXIIa-S)_s])) - ((ka_37*[(FXII-S)_s]*[(PKa-HK-S)_s])-(kd_37*[(FXII-PKa-HK-S)_s])) - ((ka_38*[(FXII-S)_s]*[(PKa-cHK-S)_s])-(kd_38*[(FXII-PKa-cHK-S)_s])))
22	d([(PKa-C1inh)_v])/dt = 1/(-((ka_16*[(PKa-C1inh)_v]*cHK_v)-(kd_16*[(PKa-C1inh-cHK)_v])) - ((ka_17*[(PKa-C1inh)_v]*[(cHK-S)_s])-(kd_17*[(PKa-C1inh-cHK-S)_s])) - ((ka_46*[(PKa-C1inh)_v]*[(HK-S)_s])-(kd_46*[(PKa-C1inh-HK-S)_s])) - ((ka_49*[(PKa-C1inh)_v]*HK_v)-(kd_49*[(PKa-C1inh-HK)_v])) + ((ka_62*PKa_v*C1inh_v)))
23	d([βFXIIa_v])/dt = 1/(((kcat_5*[(αFXIIa-S-αFXIIa-S)_s])) - ((ka_31*PK_v*[βFXIIa_v])-(kd_31*[(PK-βFXIIa)_v])) + ((kcat_31*[(PK-βFXIIa)_v])) - ((ka_32*[PK-HK)_v]*[βFXIIa_v])-(kd_32*[(PK-HK-βFXIIa)_v])) + ((kcat_32*[(PK-HK-βFXIIa)_v])) - ((ka_33*[(PK-cHK)_v]*[βFXIIa_v])-(kd_33*[(PK-cHK-βFXIIa)_v])) + ((kcat_33*[(PK-cHK-βFXIIa)_v])) + ((kcat_39*[(αFXIIa-S-PKa-HK-S)_s])) + ((kcat_40*[(αFXIIa-S-PKa-cHK-S)_s])) - ((ka_59*[βFXIIa_v]*C1inh_v)) - ((ka_60*[βFXIIa_v]*[α2AP_v])) - ((ka_61*[βFXIIa_v]*AT_v)))
24	d([(βFXIIa- AT)_v])/dt = 1/(((ka_61*[βFXIIa_v]*AT_v)))
25	d([α2AP_v])/dt = 1/(-((ka_53*[αFXIIa_v]*[α2AP_v])) - ((ka_54*[(αFXIIa-S)_s]*[α2AP_v])) - ((ka_60*[βFXIIa_v]*[α2AP_v])))
26	d([(βFXIIa- α2AP)_v])/dt = 1/(((ka_60*[βFXIIa_v]*[α2AP_v])))
27	d([(βFXIIa-C1inh)_v])/dt = 1/(((ka_59*[βFXIIa_v]*C1inh_v)))
28	d(PK_v)/dt = 1/(-((ka_10*PK_v*HK_v)-(kd_10*[(PK-HK)_v])) - ((ka_11*PK_v*cHK_v)-(kd_11*[(PK-cHK)_v])) - ((ka_12*PK_v*[(HK-S)_s])-(kd_12*[(PK- HK-S)_s])) - ((ka_13*PK_v*[(cHK-S)_s])-(kd_13*[(PK-cHK-S)_s])) - ((ka_28*PK_v*[(αFXIIa-S)_s])-(kd_28*[(PK-αFXIIa-S)_s])) - ((ka_31*PK_v*[βFXIIa_v])-(kd_31*[(PK-βFXIIa)_v])))
29	d([(αFXIIa-S)_s])/dt = 1/(((ka_2*[αFXIIa_v]*S_s)-(kd_2*[(αFXIIa-S)_s])) + ((ka_3*[(FXII-S)_s])) - ((ka_4*[(FXII-S)_s]*[(αFXIIa-S)_s])-(kd_4*[(FXII-S-αFXIIa-S)_s])) + 2*((kcat_4*[(FXII-S-αFXIIa-S)_s])) - 2*((ka_5*[(αFXIIa-S)_s]*[(αFXIIa-S)_s])-(kd_5*[(αFXIIa-S-αFXIIa-S)_s])) + ((kcat_5*[(αFXIIa-S-αFXIIa-S)_s])) - ((ka_28*PK_v*[(αFXIIa-S)_s])-(kd_28*[(PK-αFXIIa-S)_s])) + ((kcat_28*[(PK-αFXIIa-S)_s])) - ((ka_29*[(PK-HK-S)_s]*[(αFXIIa-S)_s])-(kd_29*[(PK-HK-S-αFXIIa-S)_s])) + ((kcat_29*[(PK-HK-S-αFXIIa-S)_s])) - ((ka_30*[(PK-cHK-S)_s]*[(αFXIIa-S)_s])-(kd_30*[(PK-cHK-S-αFXIIa-S)_s])) + ((kcat_30*[(PK-cHK-S-αFXIIa-S)_s])) + ((kcat_37*[(FXII-PKa-HK-S)_s])) + ((kcat_38*[(FXII-PKa-cHK-S)_s])) - ((ka_39*[(αFXIIa-S)_s]*[(PKa-HK-S)_s])-(kd_39*[(αFXIIa-S-PKa-HK-S)_s])) - ((ka_40*[(αFXIIa-S)_s]*[(PKa-cHK-S)_s])-(kd_40*[(αFXIIa-S-PKa-cHK-S)_s])) - ((ka_54*[(αFXIIa-S)_s]*[α2AP_v])) - ((ka_56*[(αFXIIa-S)_s]*[α2M_v])) - ((ka_58*[(αFXIIa-S)_s]*AT_v)))
30	d([(PK-αFXIIa-S)_s])/dt = 1/(((ka_28*PK_v*[(αFXIIa-S)_s])-(kd_28*[(PK-αFXIIa-S)_s])) - ((kcat_28*[(PK-αFXIIa-S)_s])))
31	d([(PK-HK-S)_s])/dt = 1/(((ka_24*[(PK-HK)_v]*[1.5*S_s])-(kd_24*[(PK-HK-S)_s])) - ((ka_29*[(PK-HK-S)_s]*[(αFXIIa-S)_s])-(kd_29*[(PK-HK-S-αFXIIa-S)_s])) - ((ka_41*[(PK-HK-S)_s]*[(PKa-HK-S)_s])) - ((ka_43*[(PK-HK-S)_s]*[(PKa-cHK-S)_s])))
32	d([(PK-HK-S-αFXIIa-S)_s])/dt = 1/(((ka_29*[(PK-HK-S)_s]*[(αFXIIa-S)_s])-(kd_29*[(PK-HK-S-αFXIIa-S)_s])) - ((kcat_29*[(PK-HK-S-αFXIIa-S)_s])))
33	d([αFXIIa_v])/dt = 1/(-((ka_2*[αFXIIa_v]*S_s)-(kd_2*[(αFXIIa-S)_s])) + ((kcat_34*[(FXII-PKa)_v])) - ((ka_52*[αFXIIa_v]*C1inh_v)) - ((ka_53*[αFXIIa_v]*[α2AP_v])) - ((ka_55*[αFXIIa_v]*[α2M_v])) - ((ka_57*[αFXIIa_v]*AT_v)))
34	d([(FXII-S-αFXIIa-S)_s])/dt = 1/(((ka_4*[(FXII-S)_s]*[(αFXIIa-S)_s])-(kd_4*[(FXII-S-αFXIIa-S)_s])) - ((kcat_4*[(FXII-S-αFXIIa-S)_s])))
35	d([(αFXIIa-S-αFXIIa-S)_s])/dt = 1/(((ka_5*[(αFXIIa-S)_s]*[(αFXIIa-S)_s])-(kd_5*[(αFXIIa-S-αFXIIa-S)_s])) - ((kcat_5*[(αFXIIa-S-αFXIIa-S)_s])))
36	d(FXI_v)/dt = 1/(-((ka_6*FXI_v*HK_v)-(kd_6*[(FXI-HK)_v])) - ((ka_7*FXI_v*cHK_v)-(kd_7*[(FXI-cHK)_v])) - ((ka_8*FXI_v*[(HK-S)_s])-(kd_8*[(FXI-HK-S)_s])) - ((ka_9*FXI_v*[(cHK-S)_s])-(kd_9*[(FXI-cHK-S)_s])))
37	d(HK_v)/dt = 1/(-((ka_6*FXI_v*HK_v)-(kd_6*[(FXI-HK)_v])) - ((ka_10*PK_v*HK_v)-(kd_10*[(PK-HK)_v])) - ((ka_20*HK_v*[1.5*S_s])-(kd_20*[(HK-S)_s])) - ((ka_48*PKa_v*HK_v)-(kd_48*[(PKa-

	HK)_v])) - ((ka_49*[(PKa-C1inh)_v]*HK_v)-(kd_49*[(PKa-C1inh-HK)_v])) - ((ka_50*[(PKa-AT)_v]*HK_v)-(kd_50*[(PKa-AT-HK)_v])))
38	d([(FXI-HK)_v])/dt = 1/(((ka_6*FXI_v*HK_v)-(kd_6*[(FXI-HK)_v])) - ((ka_22*[(FXI-HK)_v]*[1.5*S_s])-(kd_22*[(FXI-HK-S)_s])))
39	d(cHK_v)/dt = 1/(-((ka_7*FXI_v*cHK_v)-(kd_7*[(FXI-cHK)_v])) - ((ka_11*PK_v*cHK_v)-(kd_11*[(PK-cHK)_v])) - ((ka_14*PKa_v*cHK_v)-(kd_14*[(PKa-cHK)_v])) - ((ka_16*[(PKa-C1inh)_v]*cHK_v)-(kd_16*[(PKa-C1inh-cHK)_v])) - ((ka_18*[(PKa-AT)_v]*cHK_v)-(kd_18*[(PKa-AT-cHK)_v])) - ((ka_21*cHK_v*[1.5*S_s])-(kd_21*[(cHK-S)_s])))
40	d([(FXI-cHK)_v])/dt = 1/(((ka_7*FXI_v*cHK_v)-(kd_7*[(FXI-cHK)_v])) - ((ka_23*[(FXI-cHK)_v]*[1.5*S_s])-(kd_23*[(FXI-cHK-S)_s])))
41	d([(HK-S)_s])/dt = 1/(-((ka_8*FXI_v*[(HK-S)_s])-(kd_8*[(FXI-HK-S)_s])) - ((ka_12*PK_v*[(HK-S)_s])-(kd_12*[(PK- HK-S)_s])) + ((ka_20*HK_v*[1.5*S_s])-(kd_20*[(HK-S)_s])) - ((ka_45*PKa_v*[(HK-S)_s])-(kd_45*[(PKa-HK-S)_s])) - ((ka_46*[(PKa-C1inh)_v]*[(HK-S)_s])-(kd_46*[(PKa-C1inh-HK-S)_s])) - ((ka_47*[(PKa-AT)_v]*[(HK-S)_s])-(kd_47*[(PKa-AT-HK-S)_s])))
42	d([(FXI-HK-S)_s])/dt = 1/(((ka_8*FXI_v*[(HK-S)_s])-(kd_8*[(FXI-HK-S)_s])) + ((ka_22*[(FXI-HK)_v]*[1.5*S_s])-(kd_22*[(FXI-HK-S)_s])))
43	d([(cHK-S)_s])/dt = 1/(-((ka_9*FXI_v*[(cHK-S)_s])-(kd_9*[(FXI-cHK-S)_s])) - ((ka_13*PK_v*[(cHK-S)_s])-(kd_13*[(PK-cHK-S)_s])) - ((ka_15*PKa_v*[(cHK-S)_s])-(kd_15*[(PKa-cHK-S)_s])) - ((ka_17*[(PKa-C1inh)_v]*[(cHK-S)_s])-(kd_17*[(PKa-C1inh-cHK-S)_s])) - ((ka_19*[(PKa-AT)_v]*[(cHK-S)_s])-(kd_19*[(PKa-AT-cHK-S)_s])) + ((ka_21*cHK_v*[1.5*S_s])-(kd_21*[(cHK-S)_s])))
44	d([(FXI-cHK-S)_s])/dt = 1/(((ka_9*FXI_v*[(cHK-S)_s])-(kd_9*[(FXI-cHK-S)_s])) + ((ka_23*[(FXI-cHK)_v]*[1.5*S_s])-(kd_23*[(FXI-cHK-S)_s])))
45	d([(PK-HK)_v])/dt = 1/(((ka_10*PK_v*HK_v)-(kd_10*[(PK-HK)_v])) - ((ka_24*[(PK-HK)_v]*[1.5*S_s])-(kd_24*[(PK-HK-S)_s])))
46	d([(PK-cHK)_v])/dt = 1/(((ka_11*PK_v*cHK_v)-(kd_11*[(PK-cHK)_v])) - ((ka_25*[(PK-cHK)_v]*[1.5*S_s])-(kd_25*[(PK-cHK-S)_s])) - ((ka_33*[(PK-cHK)_v]*[βFXIIa_v])-(kd_33*[(PK-cHK-βFXIIa)_v])))
47	d([(PK- HK-S)_s])/dt = 1/(((ka_12*PK_v*[(HK-S)_s])-(kd_12*[(PK- HK-S)_s])))
48	d([(PK-cHK-S)_s])/dt = 1/(((ka_13*PK_v*[(cHK-S)_s])-(kd_13*[(PK-cHK-S)_s])) + ((ka_25*[(PK-cHK)_v]*[1.5*S_s])-(kd_25*[(PK-cHK-S)_s])) - ((ka_30*[(PK-cHK-S)_s]*[(αFXIIa-S)_s])-(kd_30*[(PK-cHK-S-αFXIIa-S)_s])) - ((ka_42*[(PK-cHK-S)_s]*[(PKa-HK-S)_s])) - ((ka_44*[(PK-cHK-S)_s]*[(PKa-cHK-S)_s])))
49	d([(αFXIIa-S-AT)_s])/dt = 1/(((ka_58*[(αFXIIa-S)_s]*AT_v)))
50	d([αFXIIa-AT_v])/dt = 1/(((ka_57*[αFXIIa_v]*AT_v)))
51	d([(FXII-PKa)_v])/dt = 1/(((ka_34*FXII_v*PKa_v)-(kd_34*[(FXII-PKa)_v])) - ((kcat_34*[(FXII-PKa)_v])))
52	d([(FXII-PKa-HK)_v])/dt = 1/(((ka_35*FXII_v*[(PKa-HK)_v])-(kd_35*[(FXII-PKa-HK)_v])) - ((kcat_35*[(FXII-PKa-HK)_v])) + ((ka_36*FXII_v*[(PKa-HK)_v])-(kd_36*[(FXII-PKa-HK)_v])) - ((kcat_36*[(FXII-PKa-HK)_v])))
53	d([αFXII_v])/dt = 1/(((kcat_35*[(FXII-PKa-HK)_v])) + ((kcat_36*[(FXII-PKa-HK)_v])))
54	d([(FXII-PKa-HK-S)_s])/dt = 1/(((ka_37*[(FXII-S)_s]*[(PKa-HK-S)_s])-(kd_37*[(FXII-PKa-HK-S)_s])) - ((kcat_37*[(FXII-PKa-HK-S)_s])))
55	d([(FXII-PKa-cHK-S)_s])/dt = 1/(((ka_38*[(FXII-S)_s]*[(PKa-cHK-S)_s])-(kd_38*[(FXII-PKa-cHK-S)_s])) - ((kcat_38*[(FXII-PKa-cHK-S)_s])))
56	d([(PK-cHK-βFXIIa)_v])/dt = 1/(((ka_33*[(PK-cHK)_v]*[βFXIIa_v])-(kd_33*[(PK-cHK-βFXIIa)_v])) - ((kcat_33*[(PK-cHK-βFXIIa)_v])))
57	d([(PK-HK-βFXIIa)_v])/dt = 1/(((ka_32*[PK-HK)_v]*[βFXIIa_v])-(kd_32*[(PK-HK-βFXIIa)_v])) - ((kcat_32*[(PK-HK-βFXIIa)_v])))
58	d([PK-HK)_v])/dt = 1/(-((ka_32*[PK-HK)_v]*[βFXIIa_v])-(kd_32*[(PK-HK-βFXIIa)_v])))
59	d([(PK-βFXIIa)_v])/dt = 1/(((ka_31*PK_v*[βFXIIa_v])-(kd_31*[(PK-βFXIIa)_v])) - ((kcat_31*[(PK-βFXIIa)_v])))
60	d([(PK-cHK-S-αFXIIa-S)_s])/dt = 1/(((ka_30*[(PK-cHK-S)_s]*[(αFXIIa-S)_s])-(kd_30*[(PK-cHK-S-αFXIIa-S)_s])) - ((kcat_30*[(PK-cHK-S-αFXIIa-S)_s])))
61	d([(PKa-AT-cHK-S)_s])/dt = 1/(((ka_19*[(PKa-AT)_v]*[(cHK-S)_s])-(kd_19*[(PKa-AT-cHK-S)_s])))
62	d([(PKa-AT-cHK)_v])/dt = 1/(((ka_18*[(PKa-AT)_v]*cHK_v)-(kd_18*[(PKa-AT-cHK)_v])))
63	d([(αFXIIa-S-PKa-cHK-S)_s])/dt = 1/(((ka_40*[(αFXIIa-S)_s]*[(PKa-cHK-S)_s])-(kd_40*[(αFXIIa-S-PKa-cHK-S)_s])) - ((kcat_40*[(αFXIIa-S-PKa-cHK-S)_s])))
64	d(BK_v)/dt = 1/(((kcat_45*[(PKa-HK-S)_s])) + ((kcat_48*[(PKa-HK)_v])) - ((ka_51*BK_v)))

65	d([(PKa-AT-HK-S)_s])/dt = 1/(((ka_47*[(PKa-AT)_v]*[(HK-S)_s])-(kd_47*[(PKa-AT-HK-S)_s])))
66	d([(PKa-AT-HK)_v])/dt = 1/(((ka_50*[(PKa-AT)_v]*HK_v)-(kd_50*[(PKa-AT-HK)_v])))
67	d(BK_deg_v)/dt = 1/(((ka_51*BK_v)))
68	d([αFXIIa-C1inh_v])/dt = 1/(((ka_52*[αFXIIa_v]*C1inh_v)))
69	d([αFXIIa- α2AP_v])/dt = 1/(((ka_53*[αFXIIa_v]*[α2AP_v])))
70	d([(αFXIIa-S-α2AP)_s])/dt = 1/(((ka_54*[(αFXIIa-S)_s]*[α2AP_v])))
71	d([αFXIIa-α2M_v])/dt = 1/(((ka_55*[αFXIIa_v]*[α2M_v])))
72	d([(αFXIIa-S-α2M)_s])/dt = 1/(((ka_56*[(αFXIIa-S)_s]*[α2M_v])))
73	d([(αFXIIa-S-PKa-HK-S)_s])/dt = 1/(((ka_39*[(αFXIIa-S)_s]*[(PKa-HK-S)_s])-(kd_39*[(αFXIIa-S-PKa-HK-S)_s])) - ((kcat_39*[(αFXIIa-S-PKa-HK-S)_s])))
