# Supplementary material for: A mechanistic model of in vitro plasma activation to evaluate therapeutic kallikrein-kinin system inhibitors
Source: PLoS Comput Biol. 2024 Nov 4;20(11):e1012552. doi: 10.1371/journal.pcbi.1012552 (PMC11563367; doi:10.1371/journal.pcbi.1012552)
Supplement: S3 Table — Surface-bound species indicated by a subscript ‘s’. It is important to note that the binding affinity of CSL312 to FXII zymogen, as measured in-house via surface plasmon resonance (SPR), may not be accurate. This uncertainty primarily arises from challenges in obtaining pure FXII zymogen. The presence of small amounts of FXIIa, known to have a significantly higher affinity for CSL312, may result in an overestimation of the zymogen binding affinity. To assess the impact of this uncertainty on BK inhibition, we conducted simulations using an order of magnitude lower affinity of CSL312 for the FXII zymogen. The results indicated a negligible difference in BK generation levels. (PDF) [file pcbi.1012552.s004.pdf]

	Reaction	k_a ($M^{-1}s^{-1}$)	k_d (s^{-1})	Ref
73	FXII$_v$ + CSL312$_v$ <-> (FXII-CSL312)$_v$	$1\text{x}10^6$	$5.7\text{x}10^{-2}$	Internal report
74	(FXII-S)$_s$ + CSL312$_v$ <-> (FXII-S-CSL312)$_s$			
75	αXIIa$_v$ + CSL312$_v$ <-> (αXIIa-CSL312)$_v$	$6\text{x}10^5$	$8.5\text{x}10^{-5}$	
76	(αXIIa-S)$_s$ + CSL312$_v$ <-> (αXIIa-S-CSL312)$_s$			
77	βFXIIa$_v$ + CSL312$_v$ <-> (βFXIIa-CSL312)$_v$			
78	PKa$_v$ + DX88$_v$ <-> (PKa-DX88)$_v$	$2\text{x}10^6$	$2\text{x}10^{-5}$	[1]

Reference

1. Levy JH, O'Donnell PS. The therapeutic potential of a kallikrein inhibitor for treating hereditary angioedema. Expert Opin Inv Drug. 2006;15(9):1077–90.
