# Supplementary figures and images for: A mechanistic model of in vitro plasma activation to evaluate therapeutic kallikrein-kinin system inhibitors
Source: PLoS Comput Biol. 2024 Nov 4;20(11):e1012552. doi: 10.1371/journal.pcbi.1012552 (PMC11563367; doi:10.1371/journal.pcbi.1012552)

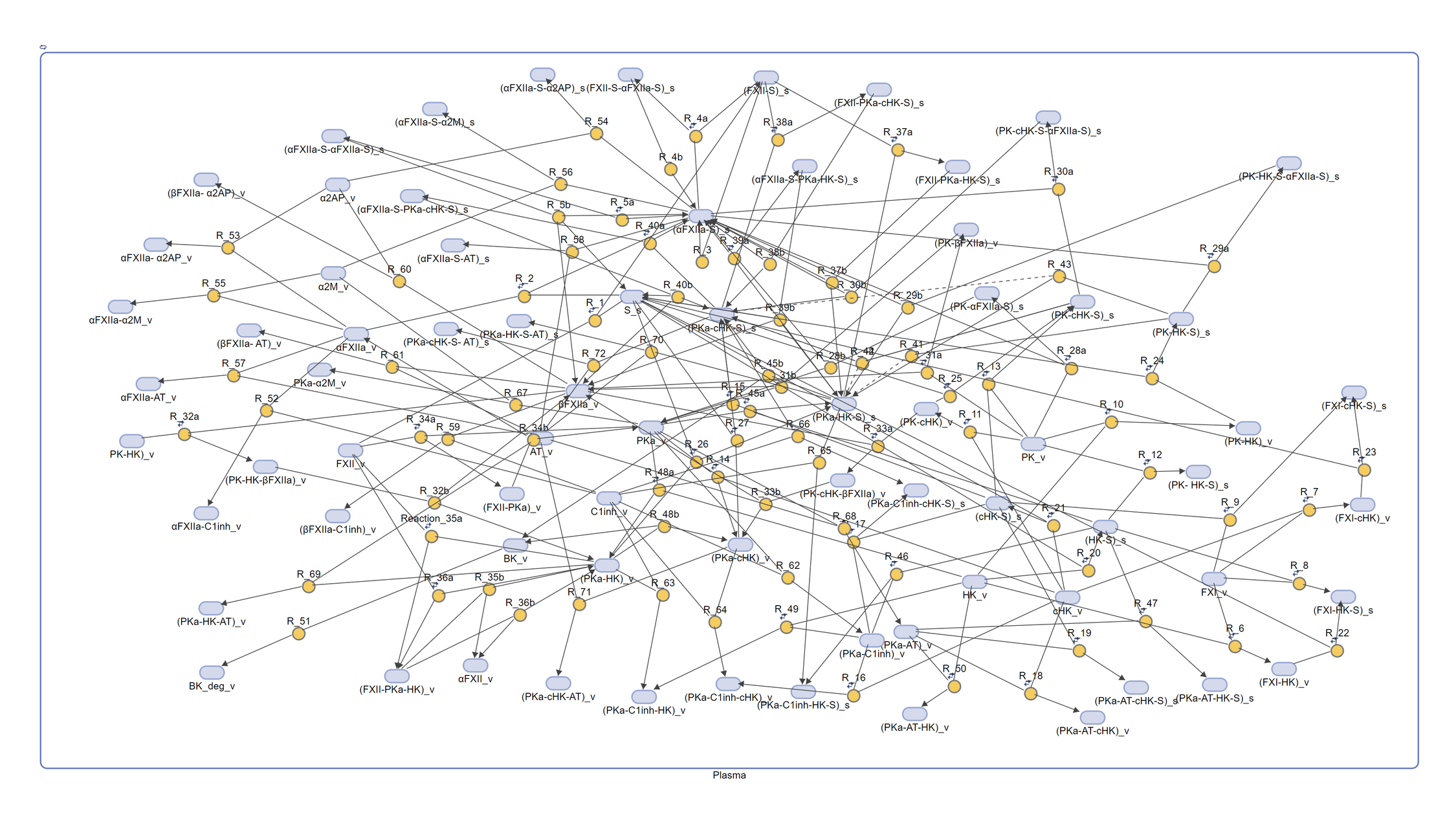

Supplement: S1 Fig — FXII: factor FXII, αFXIIa: two-chain activated factor FXII, βFXIIa: single-chain activated FXII, FXI: factor FXI, PK: prekallikrein, PKa: kallikrein, HK: high molecular weight kininogen, cHK: cleaved high molecular weight kininogen, BK: bradykinin, α2M: α2-macroglobulin, C1inh: C1 esterase inhibitor. Surface-bound species are indicated by subscript ‘s’, and solution-phase reactions are indicated by subscript ‘v’. Reaction numbers correspond to those in Table 1. (TIF) [file pcbi.1012552.s005.tif]

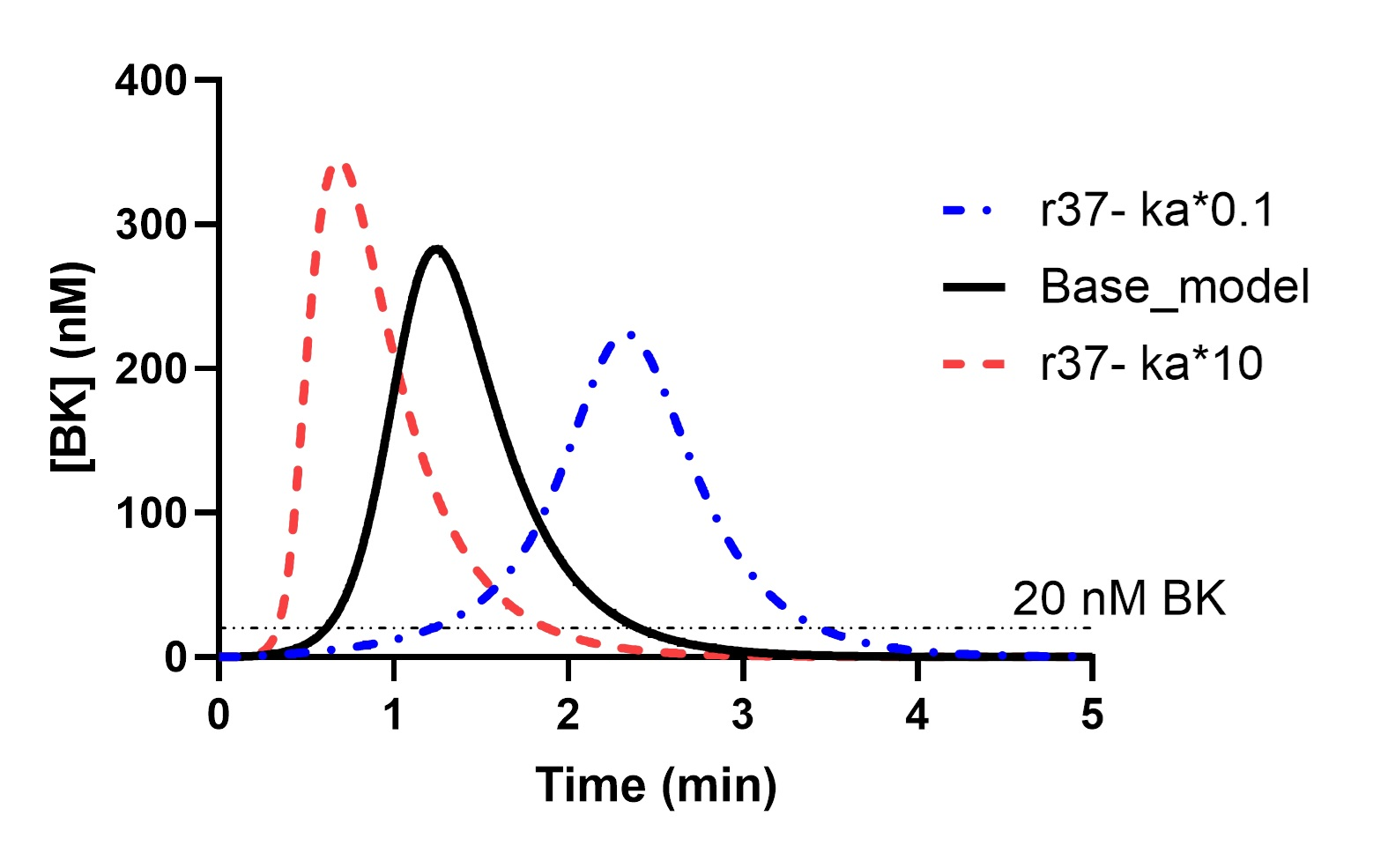

Supplement: S2 Fig — The activation rate of r37 was varied from 10% to 1000% of the baseline value, and the corresponding variations in BK levels were determined. (TIF) [file pcbi.1012552.s006.tif]

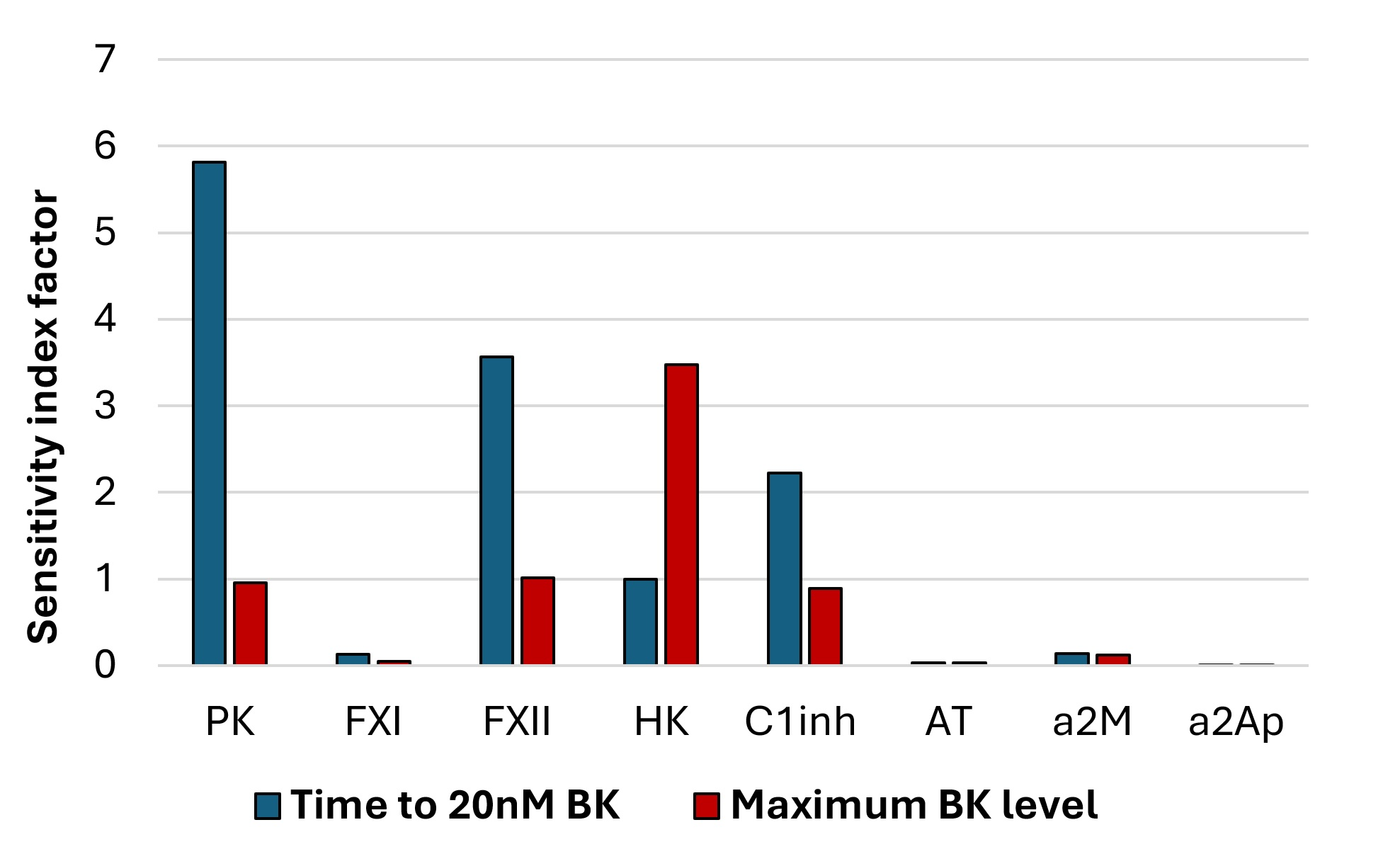

Supplement: S3 Fig — Each initial concentration was varied from 10% to 1000% of the baseline value, and the effects on the time required to generate 20 nM of BK, as well as the maximum BK level, were determined. The sensitivity index factor was calculated by dividing the variations in the time to generate 20 nM of BK or in the maximum BK level by their corresponding baseline values. (TIF) [file pcbi.1012552.s007.tif]

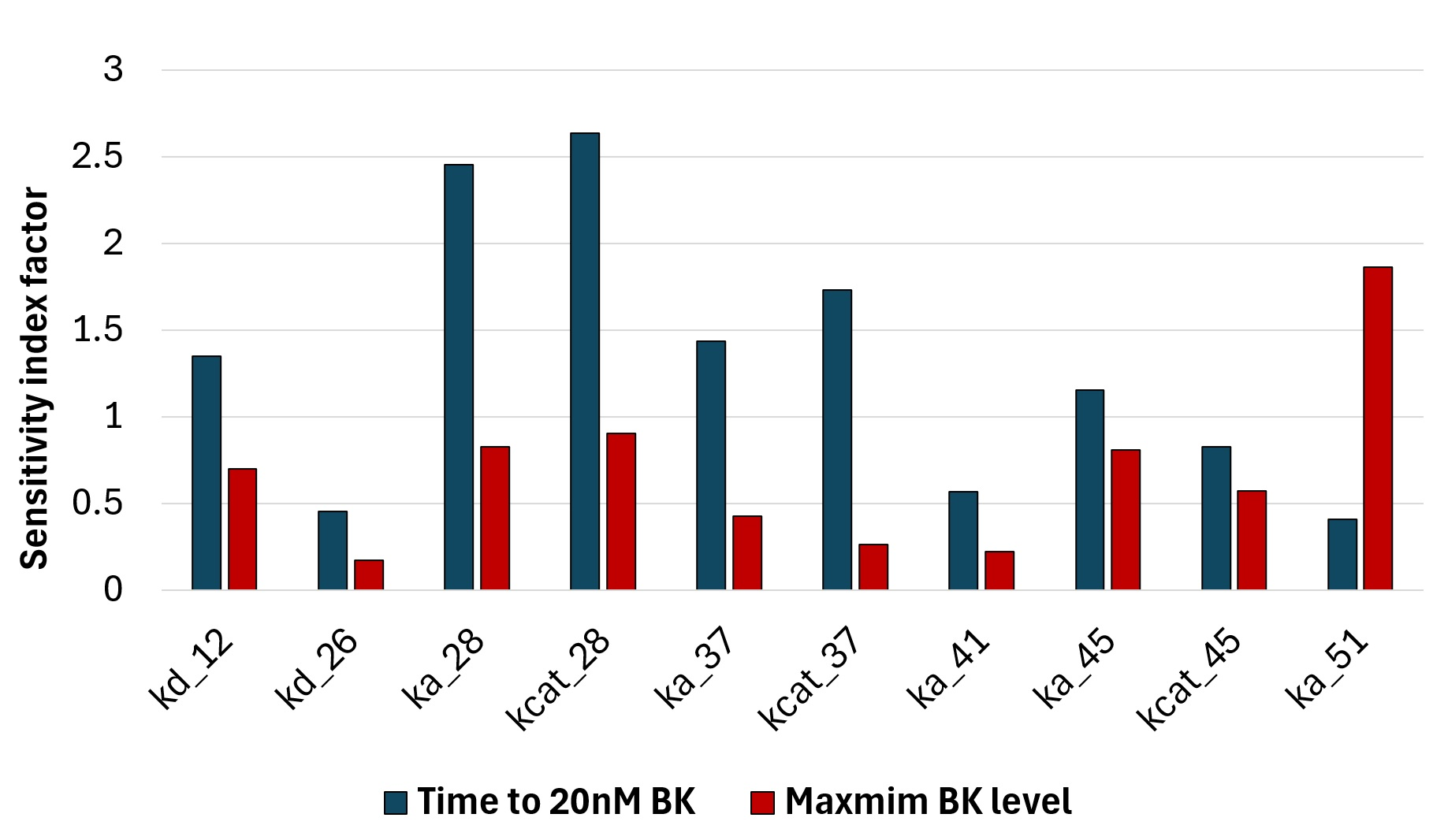

Supplement: S4 Fig — Each kinetic parameter was individually varied from 10% to 1000% of the baseline value, and the effects on the time required to generate 20 nM of BK, as well as the maximum BK level, were determined. The sensitivity index factor was calculated by dividing the variations in the time to generate 20 nM of BK or in the maximum BK level by their corresponding baseline values. This factor was depicted for the ten most sensitive parameters. (TIF) [file pcbi.1012552.s008.tif]

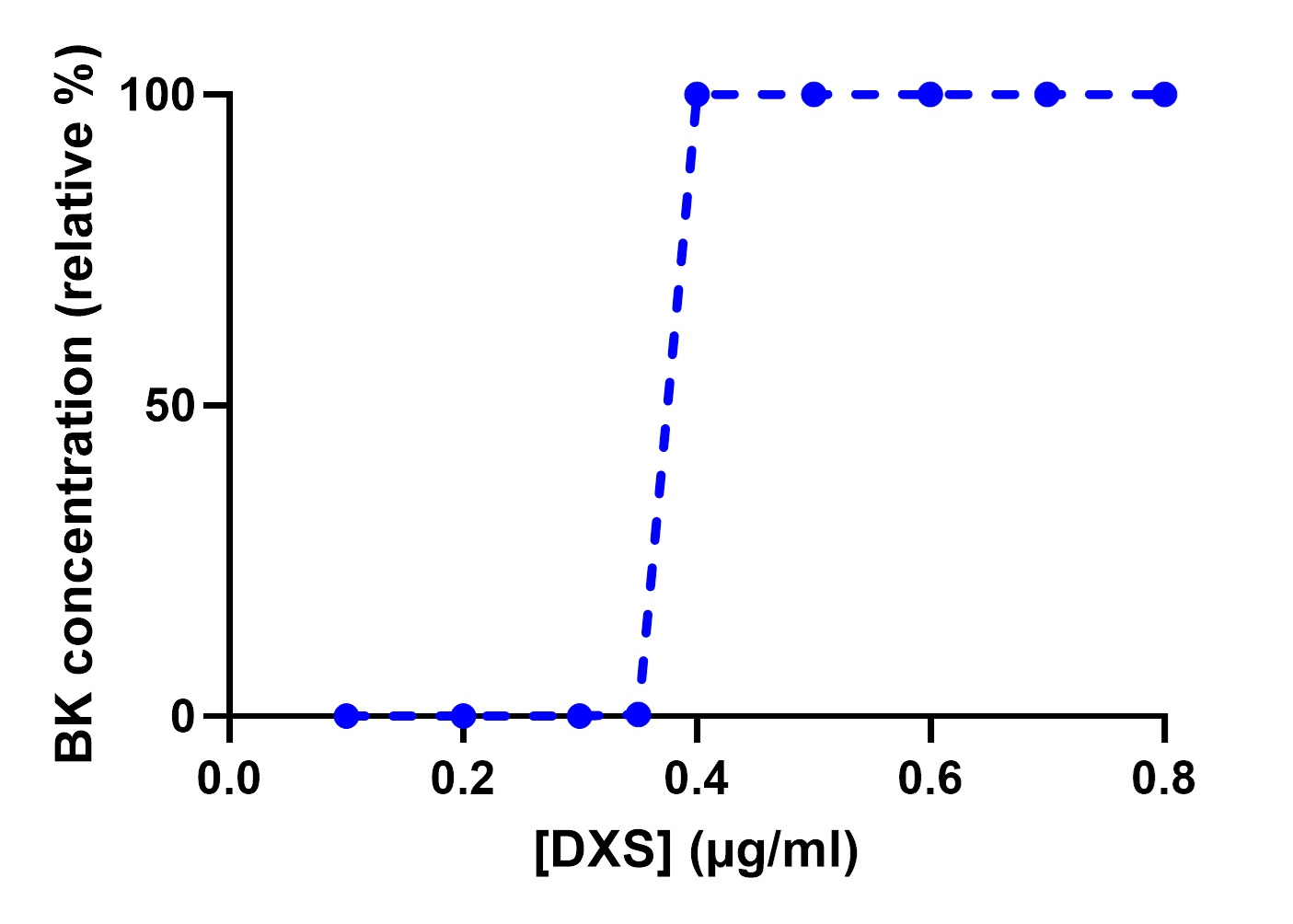

Supplement: S5 Fig — A stability zone exists at low concentrations of DXS, with no significant BK generation occurring. When the concentration of DXS exceeds a certain threshold, the steady state no longer exists, and complete cleavage of HK is observed. (TIF) [file pcbi.1012552.s009.tif]

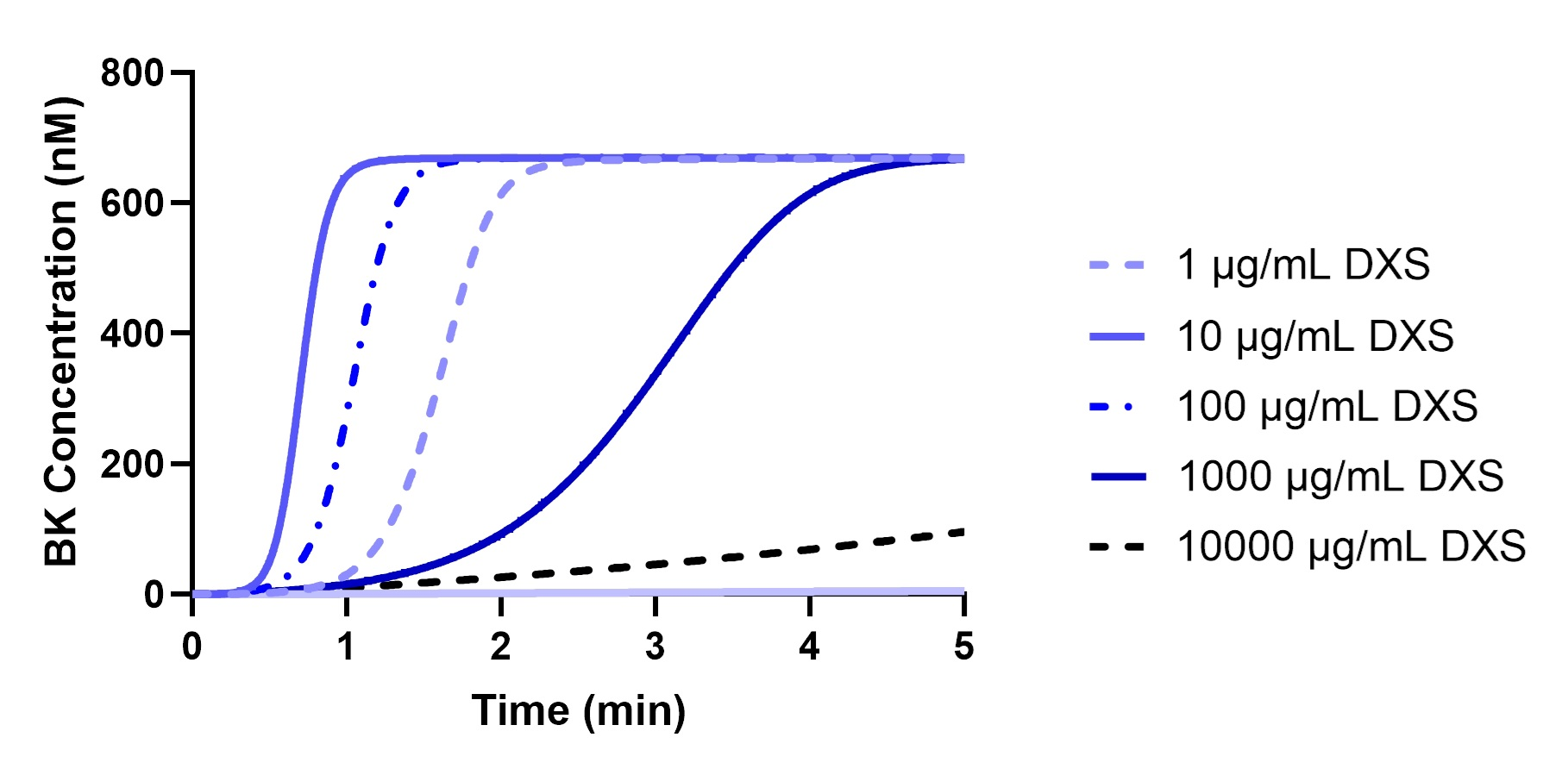

Supplement: S6 Fig — The modelling results are consistent with our experimental data showing that at 3 minutes after the addition of 1, 10, and 100 μg/ml DXS, the level of BK is the same and maximal. (TIF) [file pcbi.1012552.s010.tif]

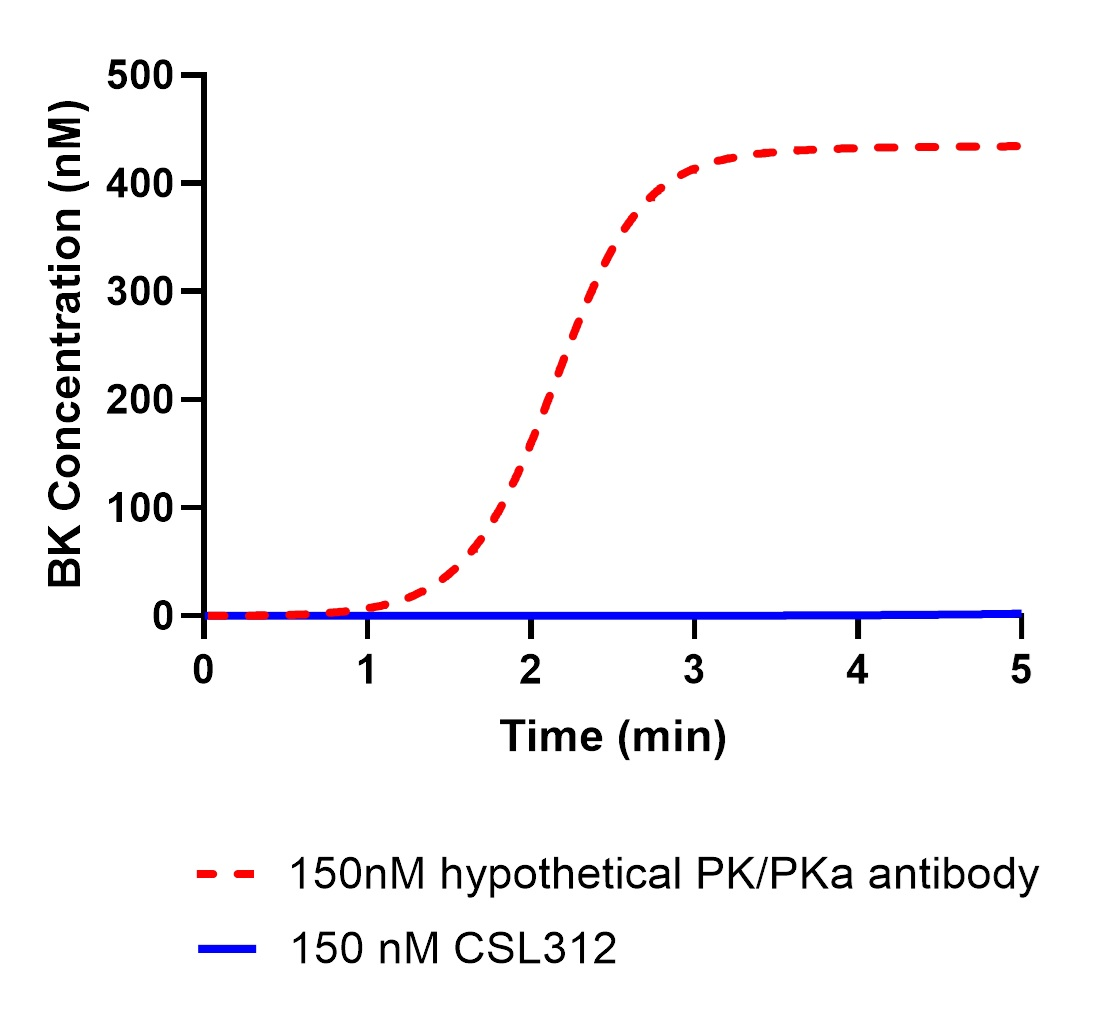

Supplement: S7 Fig — The concentration of DXS is 100μg/ml and the plasma dilution factor was 0.65. (TIF) [file pcbi.1012552.s011.tif]
